# Creation of versatile cloning platforms for transgene expression and dCas9-based epigenome editing

**DOI:** 10.1093/nar/gky1286

**Published:** 2018-12-27

**Authors:** Jonathan M Haldeman, Amanda E Conway, Michelle E Arlotto, Dorothy H Slentz, Deborah M Muoio, Thomas C Becker, Christopher B Newgard

**Affiliations:** 1 Sarah W. Stedman Nutrition and Metabolism Center, Duke Molecular Physiology Institute, Duke University Medical Center, Durham, NC 27701, USA; 2 Department of Pharmacology and Cancer Biology, Duke University Medical Center, Durham, NC 27701, USA; 3 Epigenetics & Stem Cell Biology Laboratory, National Institute of Environmental Health Sciences, Research Triangle Park, NC 27709, USA; 4 Department of Medicine, Duke University Medical Center, Durham, NC 27701, USA

## Abstract

Genetic manipulation via transgene overexpression, RNAi, or Cas9-based methods is central to biomedical research. Unfortunately, use of these tools is often limited by vector options. We have created a modular platform (pMVP) that allows a gene of interest to be studied in the context of an array of promoters, epitope tags, conditional expression modalities, and fluorescent reporters, packaged in 35 custom destination vectors, including adenovirus, lentivirus, PiggyBac transposon, and Sleeping Beauty transposon, in aggregate >108,000 vector permutations. We also used pMVP to build an epigenetic engineering platform, pMAGIC, that packages multiple gRNAs and either Sa-dCas9 or x-dCas9(3.7) fused to one of five epigenetic modifiers. Importantly, via its compatibility with adenoviral vectors, pMAGIC uniquely enables use of dCas9/LSD1 fusions to interrogate enhancers within primary cells. To demonstrate this, we used pMAGIC to target Sa-dCas9/LSD1 and modify the epigenetic status of a conserved enhancer, resulting in altered expression of the homeobox transcription factor PDX1 and its target genes in pancreatic islets and insulinoma cells. In sum, the pMVP and pMAGIC systems empower researchers to rapidly generate purpose-built, customized vectors for manipulation of gene expression, including via targeted epigenetic modification of regulatory elements in a broad range of disease-relevant cell types.

## INTRODUCTION

A core strategy for biomedical research is to genetically manipulate specific components of complex physiological systems to define regulatory mechanisms and disease-causing pathways. Such strategies can be hampered by limitations imposed by current systems for delivery of transgenes or gene suppressors into specific cell-types central to disease etiology. The pancreatic islets of Langerhans serve as a case in point. Islets are complex, spherical micro-organs comprised of five distinct endocrine cell types that participate in metabolic fuel homeostasis, mainly via the production and secretion of insulin (β-cells) and glucagon (α-cells). Loss of islet β-cell mass and function is central to the development of both major forms of diabetes mellitus (1). Our group was the first to demonstrate that cultured pancreatic islets could be efficiently transduced with recombinant serotype 5 adenoviruses (Ad5) (2), and since that time, Ad5 vectors have been used to study the impact of manipulation of specific genes on pancreatic islet cell function (2–6), replication (7–10), and survival (5,11). Whereas Ad5 vectors have proven to be an important tool to gain insights into an otherwise difficult model system, virus construction, especially for cell-type specific applications, is still laborious and time-consuming (8). Furthermore, the difficulty in engineering new Ad5 vectors hampers rapid adoption of new technologies and approaches, such as the recent advances in dCas9-mediated epigenetic engineering. Lastly, as experimental questions evolve throughout the course of a project, it is frequently desirable to utilize other experimental models (e.g. stable cell lines, transient expression) to obtain mechanistic insight. This pivot to a new model is often hampered by the lack of cross-vector compatibility. Here, we describe innovative modular cloning platforms that enable creation of highly customized adenovirus, expression plasmid, lentivirus, PiggyBac (PB) transposon or Sleeping Beauty (SB) transposon vectors for transgene or RNAi delivery, as well as dCas9-mediated epigenetic engineering vectors, that allow deployment of a cDNA, shRNA or epigenome editing modality in a customized gene delivery vector in three to 5 days.

**Figure 1.**
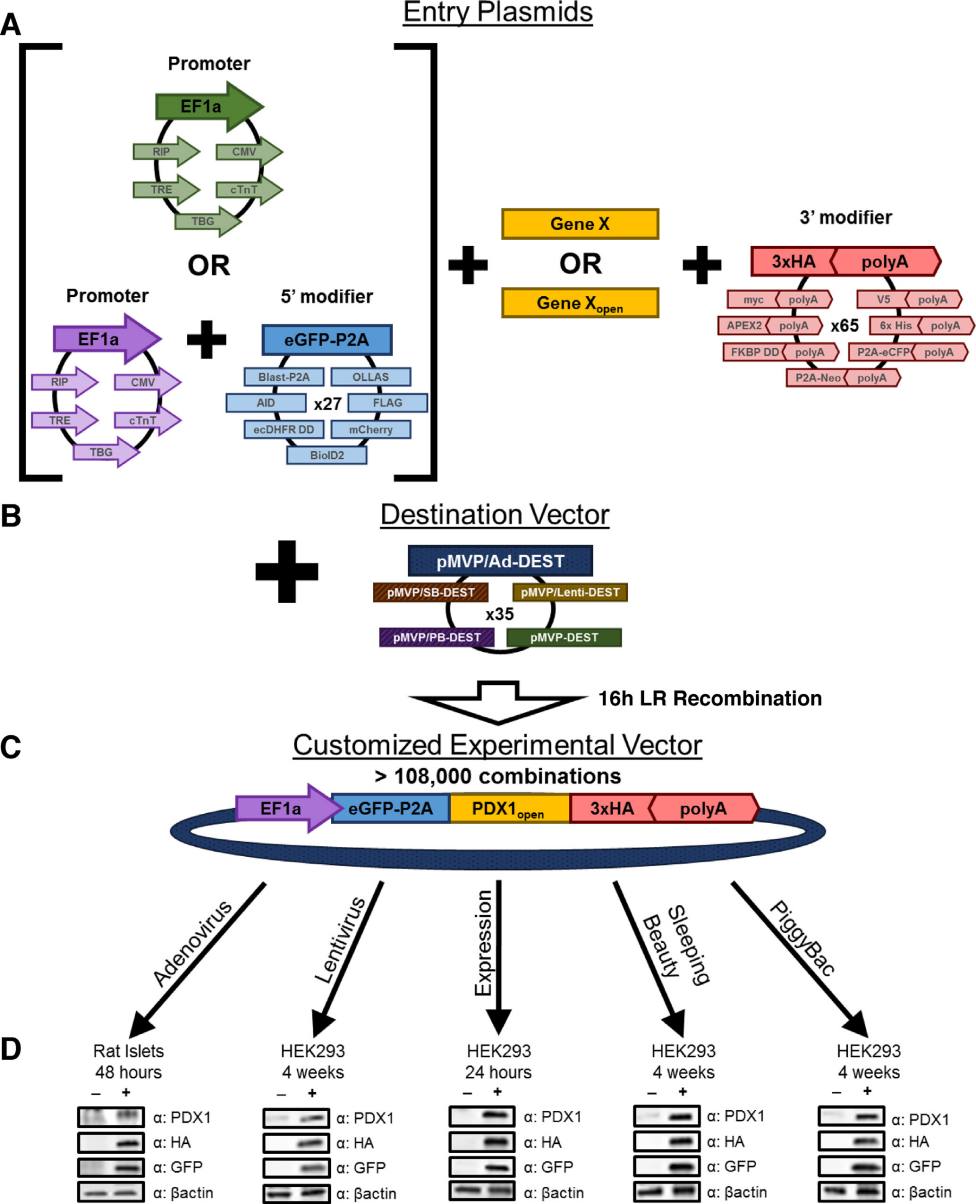
pMVP enables rapid, high-fidelity assembly of custom multicomponent transgene vectors. (**A**) The pMVP platform provides an array of Entry plasmids (pENTR) for promoters (6 options), 5′ modifiers (27 options), and 3′ modifiers (65 options) that can be partnered with a gene of interest and rapidly recombined into (**B**) an assortment of cross-compatible destination vectors (35 options) via an overnight recombination reaction to form (**C**) a customized experimental vector. In total, >108,000 unique vector permutations are possible for a gene of interest that is incorporated into pMVP. The resulting adenovirus (Ad), lentivirus (Lenti), Expression plasmid, Sleeping Beauty Transposon (SB), and PiggyBac transposon (PB) experimental vectors can be utilized in a broad variety of experimental contexts. (**D**) pMVP-derived vectors containing an EF1a promoter driving eGFP-P2A-PDX1-3xHA and either a polyA or WPRE were generated and added to primary (rat islet) or immortalized (HEK293) cells for either transient (adenovirus; expression) or stable (lentivirus, Sleeping Beauty, PiggyBac) transgene expression experiments. Cellular lysates were harvested for immunoblot analysis at the indicated times after viral transduction or plasmid transfection.

First, we created a plasmid-based modular vector platform (pMVP) utilizing MultiSite Gateway^®^ Pro (12) cloning in lieu of traditional restriction endonuclease cloning to enable rapid, high-fidelity assembly of multicomponent vectors. We designed the platform to permit user-selectable options for vector design features, including: ubiquitous or cell-type specific promoters; conditional transgene regulation; shRNA expression; different epitope tags; mammalian selection markers; and/or fluorescent reporters for tracking transduced cells. These components, along with a gene of interest, can be efficiently recombined into 35 custom destination vectors including new expanded capacity Gateway Ad5 vectors with or without fiber-modifications, lentivirus, expression plasmid, PB transposon, or SB transposon (Figure 1). Of note, our new Ad5 vectors are also engineered to facilitate further customization via insertion of a transgene cassette into the deleted E3 region or inclusion of additional fiber modifications to alter vector tropism. Altogether, pMVP establishes a system that allows a gene of interest to be rapidly incorporated into >108,000 unique combinations of purpose-built vectors with specialized functional properties to match experimental goals.

We used the pMVP framework to create a plasmid-based modular adenovirus for genomic interrogation with Cas9 (pMAGIC). The past decade has brought remarkable progress in our ability to uncover disease-associated genomic loci. Surprisingly, a large portion of these genomic elements localize outside of protein coding regions, often in areas of active epigenetic modification, suggestive of a regulatory role (13–15). These findings create a new challenge of ascribing a function to non-protein coding regulatory regions within their native cellular contexts. Recent publications have demonstrated that nuclease-dead Cas9 (dCas9) can be utilized as a scaffold, to which addition of a guide RNA (gRNA) enables promoter-specific recruitment of transcriptional and epigenetic modifiers. This recruitment has been shown to be sufficient to alter endogenous gene expression (16,17), thereby providing a viable alternative to transgene overexpression and RNAi. Furthermore, these approaches can be applied to target epigenetic modulators to discrete endogenous regulatory elements, such as enhancers, resulting in alteration of the epigenetic status and activity of the targeted element (18–20). However, these approaches are limited by the fact that the relevant epigenetic engineering tools are spread across various cloning and vector platforms rather than being aggregated in a manner that maximizes their efficient application. Furthermore, fusion of dCas9 to large effectors, such as the 2.5 kb histone demethylase LSD1, results in inserts that are too bulky to be packaged into most viral vectors; therefore, the use of Cas9/LSD1 has been restricted to a single stable cell line (19). Since LSD1 has been suggested to be the only epigenetic modifier in the dCas9 epigenetic engineering toolbox with a selective bias for repressing enhancers versus promoters (19), the inability to efficiently deliver dCas9/LSD1 fusions into cells severely limits the deployment of this important tool. To address these technological shortcomings, we designed pMAGIC to encode one of five unique epigenetic engineering proteins (VPR (17), KRAB (16,20), p300_core_ (18), LSD1 (19,21), Dnmt3a-3L (22)) fused to either nuclease-dead *Staphylococcus aureus* Cas9 (Sa-dCas9), a small *Streptococcus pyogenes* Cas9 (Sp-Cas9) ortholog with a permissive PAM sequence (NNGRRT) (23), or nuclease-dead x-Cas9(3.7) (x-dCas9(3.7)), a recently described Sp-Cas9 mutant with exceptional PAM flexibility (NG, NNG, GAT, CAA) (24). Furthermore, we addressed the issue of insert size limitation by including our new expanded-capacity and fiber-modified Gateway Ad5 vectors in the pMAGIC platform. These adenovirus vectors also allow highly efficient gene transfer, including into primary cells. Engineering of our platform in these ways allows, for the first time, efficient delivery of Sa-dCas9 or x-dCas9(3.7) fused to a ∼3 kb effector (e.g. LSD1) and multiple gRNAs into a broad array of disease-relevant cell types.

Finally, to demonstrate the ability of pMAGIC to efficiently deliver large Cas9 fusions for biological discovery, we targeted Sa-dCas9/LSD1 to a conserved enhancer element (Area IV) proposed to modulate expression of PDX1 in mature β-cells (25). PDX1 is a homeobox transcription factor that has been shown in rodents to play a critical role in islet β-cell development, identity, and function (26–29). In humans, mutations of PDX1 cause a monogenic form of diabetes (MODY4) (30) as well as permanent neonatal diabetes (31), and complete loss of PDX1 function results in pancreas agenesis (32). In addition, single-nucleotide polymorphisms (SNPs) within a 40 kb region encompassing Area IV have a significant association with fasting glucose levels (33). Using pMAGIC-derived Ad5 vectors, we demonstrate that Sa-dCas9/LSD1-mediated epigenetic modification of the Area IV element causes a significant inhibition of endogenous PDX1 expression in both the INS1 832/13 rat insulinoma cell line and in primary rat pancreatic islets. Importantly, the decrease in PDX1 is sufficient to alter expression of a set of classical PDX1 target genes, demonstrating a crucial role of the chromatin state of this region for maintaining PDX1 gene expression and β-cell identity. These results constitute an important proof-of-concept highlighting the ability of the pMAGIC platform to enable LSD1-mediated modification of enhancers in cells that are otherwise very difficult to manipulate.

## MATERIALS AND METHODS

### Cell culture and reagents

INS1-derived 832/13 rat insulinoma cells and any derivatives were cultured as previously described (34). For SB-mediated cell line creation, TransIT-LT1 (Mirus Bio, Madison, WI) was used for co-transfection of INS1 832/13 cells with a 1:10 ratio of expression plasmid for the transposase SB100X (Addgene 34879 (35)) and the pMVP-derived SB transposon vector. After 48 h, cells were subjected to blasticidin selection and maintained as the polyclone 1367/pc. Auxin (indole-3-acetic acid) was added to 1367/pc cells and harvested as indicated. For induction studies, doxycycline (dox), trimethoprim (TMP), or Shld1 (Takara, Mountain View, CA, USA) were added to cells 24 h after adenoviral transduction and harvested at indicated timepoints. Pancreatic islets were isolated from male Wistar rats weighing ∼300 g under a protocol approved by the Duke University Institutional Animal Care and Use Committee and cultured as previously described (9). Mouse C2C12 cells (ATCC, Manassas, VA, USA) were grown in DMEM (low glucose, 10% heat inactivated FBS, 50 μg/ml Gentamycin), trypsinized with 0.05% Trypsin and plated on Collagen I coated multi-well plates (Corning, Corning, NY). When cells were 95% confluent, medium was replaced with Differentiation media (DMEM, 2% heat inactivated horse serum, 50 μg/ml gentamycin, 100 μM Carnitine). Media was changed daily and cells were transduced with adenovirus on differentiation day 3. HEK293FT cells were maintained in DMEM media (high glucose, 10% FBS, 0.1 mM MEM NEAA, 6 mM l-glutamine, 1 mM MEM Na pyruvate). For lentivirus production, TransIT-LT1 (Mirus) was used for co-transfection of equimolar amounts of the pMVP/Lenti vector, pMD2.6, and psPAX2 (Addgene 12259 and 12260, a gift from Didier Trono). Lysates were harvested 48 h after transfection and filtered through a 0.45 μm filter before cell transduction. HEK293 cells were maintained in DMEM (high glucose, 10% FBS). For adenovirus production, 100 units/ml of penicillin and streptomycin were added. For expression plasmid studies, the indicated plasmids were transfected into HEK293 cells using TransIT-LT1 (Mirus Bio). For dCas9 transcriptional activation, equimolar amounts of dCas9 vectors were used, with plasmid JH601 used to normalize DNA mass for transfection. Cell images were captured using a Zeiss Axiovert S100 (Zeiss, Thornwood, NY, USA). For SB- and PB-mediated cell line creation in HEK293 cells, TransIT-LT1 (Mirus Bio) was used for co-transfection of corresponding transposon/transposase plasmids at a ratio of 1:10 (SB) or 1:5 (PB). HEK293 cells were transduced with lentivirus lysates in the presence of 10 μg/ml polybrene. After 48h, cells were subjected to blasticidin selection and maintained as the polyclones 1405/pc (SB), 1410/pc (PB), or 1409/pc (lentivirus). A single cell suspension of HEK293 cells or the resulting stable cell lines in FACS buffer (PBS pH 7.4, 1% FBS, 20 mM HEPES, 2.5 mM glucose, 100 units/ml penicillin and streptomycin) were evaluated for eGFP expression by live-cell flow cytometry performed on a FACSCanto II (BD Biosciences, San Jose, CA, USA) and analyzed using the FACSdiva software (BD Biosciences). Cell culture reagents were purchased from ThermoFisher Scientific (Waltham, MA, USA) unless otherwise specified. Chemical reagents were from Sigma-Aldrich (St. Louis, MO, USA) unless specified otherwise.

### Molecular biology reagents

The MultiSite Gateway Pro Plus kit, pAd/PL-DEST, pEF-DEST51 and pLenti6.1/V5-DEST were purchased from ThermoFisher. pBlueScript2SK(–) was purchased from Agilent (Santa Clara, CA, USA). Restriction enzymes, NEBuilder HiFi DNA Assembly mix, and T4 DNA ligase were purchased from NEB (Ipswich, MA, USA) with the exception of SgrD1 (ThermoFisher) and BarI (SibEnzyme, West Roxbury, MA, USA). Depending on the application, we utilized competent cells purchased from ThermoFisher (ccdB Survival, Mach1, Stbl3, TOP10, Subcloning Efficiency DH5α) or NEB (NEB10-beta, NEB 5-alpha subcloning efficiency). Oligonucleotides (Supplemental Table S7) and gBlocks (Supplemental Table S8) were purchased from IDT (Coralville, IA, USA). PCR templates used for plasmid creation either originated in our lab or were received from the following: DNASU (36) HsCD00082591 (PDX1, PDX1_open_); Addgene 68495 (37) (Sa-dCas9, Sa-dCas9-VPR); Addgene 60954 (38) (KRAB); Addgene 49042 (21) (LSD1); Addgene 61357 (18) (p300_core_); Addgene 61591 (23) (Sa-Cas9, 3xHA-bGH PolyA, 3xHA-bGH PolyA + hU6-Sa-Cas9 gRNA); Addgene 49386 (39) (APEX2); Addgene 47328 (40) (osTir1); Promega pGL4.23 (Firefly Luciferase) (Promega Corporation, Madision, WI, USA); Promega pGL4.35 (Hygromycin resistance gene) (Promega); ThermoFisher pEF-DEST51 (EF1a promoter); cTnT promoter was a gift from Brent French (41); Addgene 61593 (23) (TBG promoter); Addgene 108383 (24) (x-dCas9(3.7)); Addgene 108379 (24) (x-Cas9(3.7)).

### Gateway cloning

To generate pENTR plasmids, sequences of interest flanked by the appropriate Gateway attB sites, as listed in the MultiSite Gateway Pro manual, were either synthesized as gBlocks or PCR amplified by Platinum Taq HiFi DNA polymerase (ThermoFisher) or Q5 High-Fidelity DNA Polymerase (NEB) and subsequently purified using the QIAquick PCR Purification kit (QIAGEN, Venlo, Netherlands). BP reactions containing 15–75 ng of purified PCR product, 75 ng of the appropriate MultiSite Gateway Pro pDONR221 plasmid, TE added to 4 μl, and 1 μl of BP Clonase II (ThermoFisher) were incubated at room temperature for 1–16 h. BP reactions were stopped by incubation with Proteinase K (ThermoFisher) and used to transform Mach1, TOP10, or NEB10-beta competent cells. Single colonies were selected, cultured overnight at 37°C, and plasmid DNA was isolated using the QIAprep Miniprep kit (QIAGEN). Alternatively, a gene of interest can be inserted into NcoI/BamHI digested plasmid KJ901 (Supplemental Table S2), taking care to maintain the proper codon frame. This approach was utilized to generate a pENTR for a hyperactive PB transpose (hyPBase, (42)) from synthesized gBlocks. Entry plasmids were verified by restriction digest and inserts were sequence verified (Genewiz, South Plainfield, NJ, USA). The complete sequences for all pMVP and pMAGIC pENTR clones generated during the creation and validation of these platforms are available in Supplemental Tables S1, S2 and S5.

To generate pMVP- and pMAGIC-derived vectors, LR recombination reactions containing 5 fmol of each pENTR plasmid, 10 fmol of pDEST vector, TE added to 4 μl, and 1 μl LR Clonase II Plus (ThermoFisher), were incubated for >16 h at 25°C. For RNAi vector construction, we added 5 fmol of the recombination-ready synthesized cassette encoding the H1 promoter and shPDX1 as described (3,27,43). LR reactions were stopped by incubation with Proteinase K (ThermoFisher), and used to transform TOP10, NEB10-beta, or Stbl3 competent cells. Transformations were streaked on LB plates and incubated at 30–37°C overnight, taking care to avoid colony overgrowth. Single colonies were selected, cultured for 12–16 h at 28–37°C, and plasmid DNA was isolated using the QIAprep Miniprep kit (QIAGEN). Adenoviral vectors were verified using diagnostic restriction digests for HindIII, BsrGI, and PacI, and other vectors were verified with restriction enzymes relevant to the underlying sequence as determined by *in silico* recombination using DNASTAR Lasergene (DNASTAR, Madison, WI, USA). In our experience, a 5 ml bacterial culture yielded a sufficient quantity of vector for adenovirus production or most other downstream applications. A complete list of the 43 customized vectors, and the corresponding pENTR/pDEST components used for each vector, are provided in Supplemental Table S4.

### pMVP/Ad-DEST vectors

Custom adenovirus vectors were created as described above and in Supplemental Figure S2. NEBuilder HiFi DNA assembly was utilized to replace the SrfI/BarI fragment of pAd/PL-DEST with a gBlock containing an E3 deletion mirroring pAdEasy-1 as well as a unique SgrDI site to create pMVP/Ad-DEST. For fiber modification, published sequences for modifications were synthesized as gBlocks and assembled into pBS/Fiber digested with BglII/BstBI (RGD, pK, RGD/pK) or Age/BstBI (Ad5/35). Modified pBS/Fiber plasmids were digested with BsmBI and inserted into BarI/BstBI digested pMVP/Ad-DEST. The complete sequence for pBS/Fiber as well as sequence for the SrfI/BstBI fragment (E3-deleted region and Fiber gene) for all adenovirus vectors are listed in Supplemental Table S3.

### pMVP-DEST vectors

A Gateway cloning cassette (ThermoFisher) was ligated into EcoRV digested pBlueScript2SK(-) (creating pMVP_BS_-DEST) and we subsequently removed all non-essential plasmid components by replacing the XhoI/PsiI and SapI/SacI fragments with annealed oligonucleotides to create pMVP-DEST. We then utilized NEBuilder HiFi DNA Assembly to introduce an SV40 promoter, TK polyA signal, and mammalian selection markers for eGFP, mCherry, eCFP, blasticidin, puromycin, neomycin, hygromycin, or zeocin into XhoI digested pMVP-DEST. Complete vector sequences are listed in Supplemental Table S3.

### pMVP/SB-DEST vectors

NEBuilder HiFi DNA assembly was used to insert gBlocks containing SB ITR elements designed from Addgene 26557 (Courtesy of Perry Hackett) into the SpeI and BsaXI restriction sites located on either side of the pMVP-DEST Gateway cloning cassette to create pMVP/SB-DEST. In the same manner as for expression vectors, the mammalian selection markers for eGFP, mCherry, eCFP, blasticidin, puromycin, neomycin, hygromycin, or zeocin were inserted in the XhoI restriction site located between the attR2 Gateway site and adjacent 3′ SB ITR of pMVP/SB-DEST. SB experimental vectors can be integrated into a host cell via co-transfection with the SB100X transposase expression vector (Addgene 34879 (35)). Complete vector sequences are listed in Supplemental Table S3.

### pMVP/PB-DEST vectors

NEBuilder HiFi DNA assembly was used to insert gBlocks containing PB ITR elements designed from Addgene 63800 (17) into the SacII/SpeI and BsaXI restriction sites located on either side of the pMVP-DEST Gateway cloning cassette to create pMVP/PB-DEST. To aid in diagnostic digests, oligonucleotides encoding a BsrGI restriction site were ligated into the PciI restriction site. In the same manner as for expression vectors, the mammalian selection markers for either eGFP, mCherry, eCFP, blasticidin, puromycin, neomycin, hygromycin or zeocin were inserted in the XhoI restriction site located between the attR2 Gateway site and adjacent 3′ PB ITR of pMVP/PB-DEST. PB experimental vectors can be integrated into a host cell via co-transfection with the hyPBase transposase (42) expression vector described (KY201, Supplemental Table S4), or commercially available from System Bioscience. Complete vector sequences are listed in Supplemental Table S3.

### pMVP/Lenti-DEST vectors

Using pLenti6.3/V5-DEST as a template, a third-generation lentivirus vector devoid of a selection marker was generated by sequential replacement of the ClaI/SpeI fragment (CMV promoter) and XhoI/BlpI fragment (V5-WPRE-Blasticidin expression cassette) with annealed oligonucleotides to create pMVP/Lenti-DEST. A lentivirus vector conferring resistance to blasticidin (pMVP/Lenti/Blast-DEST) was generated by sequential replacement of the ClaI/SpeI fragment (CMV promoter) and the XhoI/SpeI fragment (V5 epitope tag and WPRE element) with annealed oligonucleotides. Next, NEBuilder HiFi DNA assembly was used replace the PmlI/BlpI fragment containing the blasticidin resistance gene with a neomycin resistance gene (pMVP/Lenti/Neo-DEST). pMVP/Lenti vectors are compatible with both second and third generation lentivirus packaging systems. Complete vector sequences are listed in Supplemental Table S3.

### gRNA cloning

Protospacer sequences targeting Area IV and the TRE promoter were selected and screened for potential off-target activity using the resources at www.Deskgen.com (44) or CRISPOR (45). Oligonucleotides containing protospacer motifs (Supplemental Table S6) were annealed and subsequently ligated for 20 minutes at room temperature with T4 DNA ligase into BsaI-digested pENTR plasmids containing the human U6 promoter (U6) and either a Sa-Cas9 gRNA sequence from Addgene 61591 (23) (IA102), plasmids derived from IA102 with an updated Sa-Cas9 gRNA (46), or plasmids containing an x-Cas9 expression cassette derived as described (47). Ligation reactions were re-digested with BsaI for 15 minutes to reduce background and transformed into Subcloning Efficiency DH5α competent cells. Resulting plasmids were sequence verified before use (Genewiz). As an alternative, protospacer oligonucleotides may also be ligated into BsaI-digested gRNA plasmids driven by the mouse U6 (mU6) promoter. It should be noted that for simplicity we used the nomenclature x-Cas9(3.7) gRNAs; however, these gRNAs and resulting expression cassettes are compatible with all Sp-Cas9 variants. A complete list of pMAGIC components containing gRNA expression cassettes (Supplemental Table S2) as well as gRNA plasmids containing Area IV and TRE target sequences (Supplemental Table S5) are provided.

### Recombinant adenovirus production

To produce recombinant adenoviruses, 1 μg of PacI digested vector was transfected with 1.5 μl of TransIT-LT1 (Mirus) into one well of a 24-well plate containing HEK293 cells that were 80% confluent. After primary lysis (7–10 days), crude adenovirus lysates were subject to freeze/thaw and further propagated in a confluent T25 flask of HEK293 cells to create a secondary lysate. For purification, six confluent 15 cm plates of HEK293 cells were transduced with secondary lysate and harvested after 40–60 h, at a point when 50–75% of cells were floating. Floating and attached cells were collected by gentle scraping, pelleted by centrifugation, resuspended in freeze/thaw buffer (FT buffer; 10 mM Tris–HCl pH 8.0; 1 mM MgCl_2_), and subsequently lysed by addition of NP40 to a final concentration of 0.5%. Lysates were cleared by centrifugation (10 min at 10 000 × g and 4°C), the supernatant was loaded on a pre-formed CsCl gradient (1.45, 1.33, 1.2 g/mL prepared in FT buffer), and centrifuged (1–3 h at 191 000 × g and 4°C) in a S100-AT6 rotor (ThermoFisher). The band containing mature adenoviral particles was isolated, desalted with Zeba Desalt spin columns (ThermoFisher), diluted 1:1 with sterile glycerol, filter sterilized, aliquoted, and stored at −80°C. Viral particles (vp) were determined by the *A*_260_ method (48) before addition of glycerol. Working stocks of purified adenoviruses were stored at −20°C for up to 6 months after first use. For experiments with crude viral lysates, the minimum dose sufficient for maximal transduction efficiency, determined by visual observation of eGFP expression, was used. For purified adenovirus experiments, cells were incubated with ∼5 × 10^8^ vp/cm^2^ overnight. Ad-siScramble and Ad-siPDX1 were described previously (27).

All recombinant adenoviruses were tested for the presence of replication competent adenoviruses (RCA) that can arise from recombination with the Ad5 E1A gene present in the HEK293 genome (49,50). This was performed using our recently developed qRT-PCR assay. Briefly, crude and purified adenoviruses were first subjected to DNase digest (QIAGEN) to remove any traces of contaminating HEK293 gDNA. Next, samples were protease treated and recombinant adenovirus gDNA isolated using QiaAmp MinElute Virus Spin Kit (QIAGEN). Each sample is then analyzed by qRT-PCR using primers for the Ad5 E1A gene (present only in RCA), Ad5 Hexon (L3) gene (present in all Ad), and human PPIB gene (signifies HEK293 gDNA contamination). These samples are analyzed alongside a standard curve generated using a plasmid (Newgard Plasmid ID JI401) that contains the PCR amplicons for Ad5 E1A and Ad5 Hexon. Dividing E1A values by Hexon values yields the concentration of E1A present in the sample. Adenovirus preparations with a concentration <1 ppm were considered to be RCA-negative. Primers used are as follows: E1A-F TATGCCAAACCTTGTACCGGAGGT; E1A-R CCGGGGTGCTCCACATAATCT; Hexon-F TTGGCGCATCCCATTCTCCAGTAA; Hexon-R ATAAAGAAGGGTGGGCTCGTCCAT; hPPIB-F CGCTTCCCCGATGAGAACTTC; hPPIB-R AGGCTGTCTTGACTGTCGTGATG.

### Measurement of RNA levels

Total RNA was isolated using the RNeasy Mini (INS1 832/13) or Micro (islets) kit (QIAGEN), and cDNA was synthesized using iScript (Bio-Rad). Real-time PCR (qRT-PCR) assays were performed using the QuantStudio 6 FLEX system and software (ThermoFisher). Taqman Probe primers (ThermoFisher): PPIB (Rn03302274_m1, housekeeping); PDX1 (Rn00755591_m1), GCG (Rn00562293_m1), MAFB (Rn00709456_m1), TSPAN8 (Rn00591756_m1), SLC2A2 (Rn00563565_m1), UCN3 (Rn02091611_s1) were used with TaqMan Fast Advanced Master Mix (ThermoFisher).

### Immunoblot analysis

Lysates used for immunoblotting were prepared in ice-cold RIPA Buffer containing HALT protease inhibitors (ThermoFisher). Clarified cell lysates were resolved on 4–15% mini-PROTEAN TGX pre-cast gels (Bio-Rad), and transferred to nitrocellulose membranes using a TransBlot Turbo (Bio-Rad, Hercules, CA, USA). Membranes were blocked with Odyssey TBS Blocking Buffer (Li-Cor, Licoln, NE, USA) and then probed with the appropriate antibodies. Primary antibodies were diluted in TBS + 1% PVP as follows: anti-PDX1, ab47267 1:5000 (Abcam, Cambridge, MA, USA); anti-PDX1, sc-14664, 1:5000 (Santa Cruz, Dallas, TX, USA) anti-Tubulin, T5326, 1:20000 (Sigma); anti-βactin, A5316, 1:5000 (Sigma); anti-eGFP, ab290, 1:5000 (Abcam); anti-HA, ab9110, 1:5000 (Abcam); anti-mCherry, ab125096, 1:2000 (Abcam) anti-OLLAS; NBP1-06713, 1:1000 (Novus, New York, NY); anti-FLAG, F3165-.2MG, 1:400 (Sigma); anti-His, 12698, 1:1000 (CST, Danvers, MA); anti-myc, 2278, 1:1000 (CST), anti-V5, MA5-15253, 1:1000 (ThermoFisher). Membranes were incubated with appropriate antibodies overnight at 4°C. Secondary antibodies (Li-Cor) were diluted 1:10 000 in Odyssey TBS Blocking Buffer (Li-Cor) with 0.2% Tween 20 added and incubated with membranes for 1 h at room temperature. Immunoblots were developed using a Li-Cor Odyssey CLx.

### Chromatin immunoprecipitation (ChIP)

INS1 832/13 (1 × 10^7^) cells were crosslinked with 1% formaldehyde for 8 min, and the reaction was quenched by the addition of glycine at a final concentration of 125 mM for 5 min. Cell pellets were washed twice with PBS and resuspended in 1 ml of cold lysis buffer A (50 mM HEPES pH 7.5, 140 mM NaCl, 1 mM EDTA, 10% glycerol, 0.5% IGEPAL CA-630, 0.25% Triton X-100, 1× protease inhibitor cocktail (PI; Roche)). After 10 min on ice, cells were pelleted and resuspended in 1 ml of cold lysis buffer B (10 mM Tris–HCl pH 8, 200 mM NaCl, 1 mM EDTA, 0.5 mM EGTA, 1× PI) for an additional 10 min. Cells were sonicated in cold lysis buffer C (10 mM Tris–HCl pH 8, 100 mM NaCl, 1 mM EDTA, 0.5 mM EGTA, 0.5% *N*-lauroylsarcosine, 1× PI) using Covaris S220 ultrasonicator to an average fragment size of 500 bp. Immunoprecipitation was performed with rabbit anti-HA antibody (C29F4, CST) or mouse anti-H3K27ac (#39685, Active Motif, Carlsbad, CA, USA) overnight at 4°C. Protein G Dynabeads (ThermoFisher) were added and rocked at 4°C for an additional 4 hr. Beads were washed with cold buffer TSE 150 (10 mM Tris–HCl pH 8, 150 mM NaCl, 2 mM EDTA, 1% Triton-X, 0.1% SDS), TSE 500 (10 mM Tris–HCl pH 8, 500 mM NaCl, 2 mM EDTA, 1% Triton-X, 0.1% SDS) and wash buffer (10 mM Tris–HCl pH 8, 1 mM EDTA, 0.25 M LiCl, 0.5% NP-40), and twice in cold TE (20 mM Tris–HCl pH 8, 2 mM EDTA), before being eluted in elution buffer (1% SDS, 0.1 M NaHCO3) at 65°C. Following RNAse A and proteinase K treatment, input and ChIP DNA were purified and analyzed using qRT-PCR. Amplification values were normalized to input. Primers are as follows: Chr15-F GCACAACAAAGAAACGGGCT; Chr15-R ATTGTCAGCTCCACCAGCTC; Area IV-F GCCAGCATTCGCTTTCCAAG; Area IV-R GTGCCCTTTACTCAGGAGTGG.

### Statistical analysis

Statistical significance was determined by two-sided, unpaired, equal-variance Student's *t*-test. A *P*-value of <0.05 was considered significant. All data is expressed as mean ± standard error of the mean (S.E.M.) of three (INS1 832/13 gene expression) or four (INS1 832/13 ChIP and RNAi, rat islet gene expression) independent experiments.

## RESULTS

### pMVP enables rapid assembly of unique, multicomponent vectors

**Figure 2.**
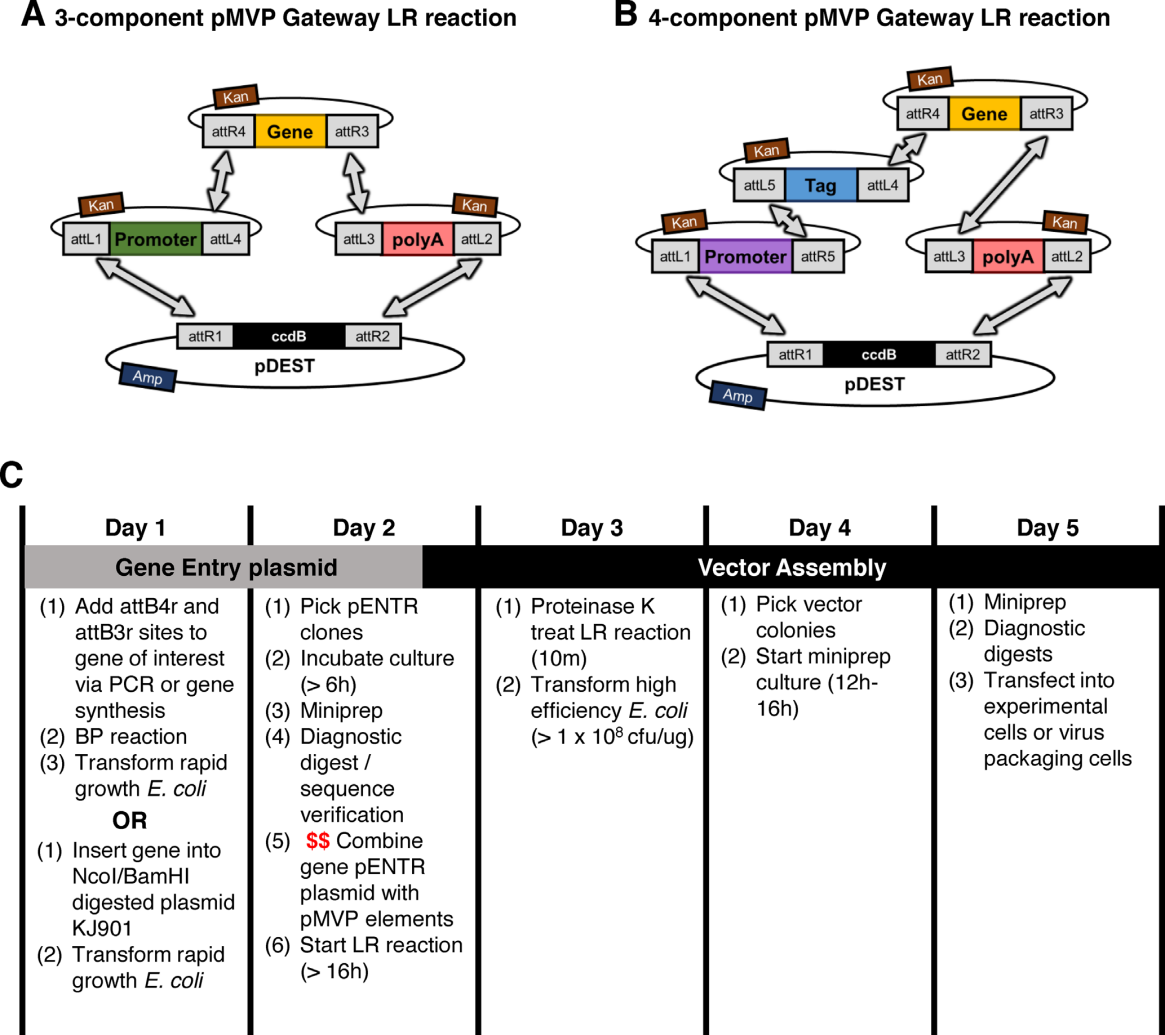
pMVP enables custom vector generation in only 3–5 days. pMVP applies Multisite Gateway Pro technologies in a manner that allows creation of an expression cassette containing a gene of interest (cloned into pENTR221 R4-R3, yellow) and Entry plasmids containing combinations of either (**A**) promoter (pENTR221 L1–L4, green) and polyA (WPRE for lentivirus; pENTR221 L3–L2, red) or (**B**) promoter (pENTR 221 L1–R5, purple), N-terminal fusion (pENTR221 L5–L4, blue), and polyA (WPRE for lentivirus; pENTR221 L3-L2, red) to be recombined into any promoterless Gateway destination vector (pDEST). (**C**) pMVP allows a gene of interest to be incorporated and utilized to create a custom vector in only 5 days. Additional vector cloning commences with the overnight recombination reaction (LR; designated by red $$ in Day 2) and can be completed within 3 days.

**Figure 3.**
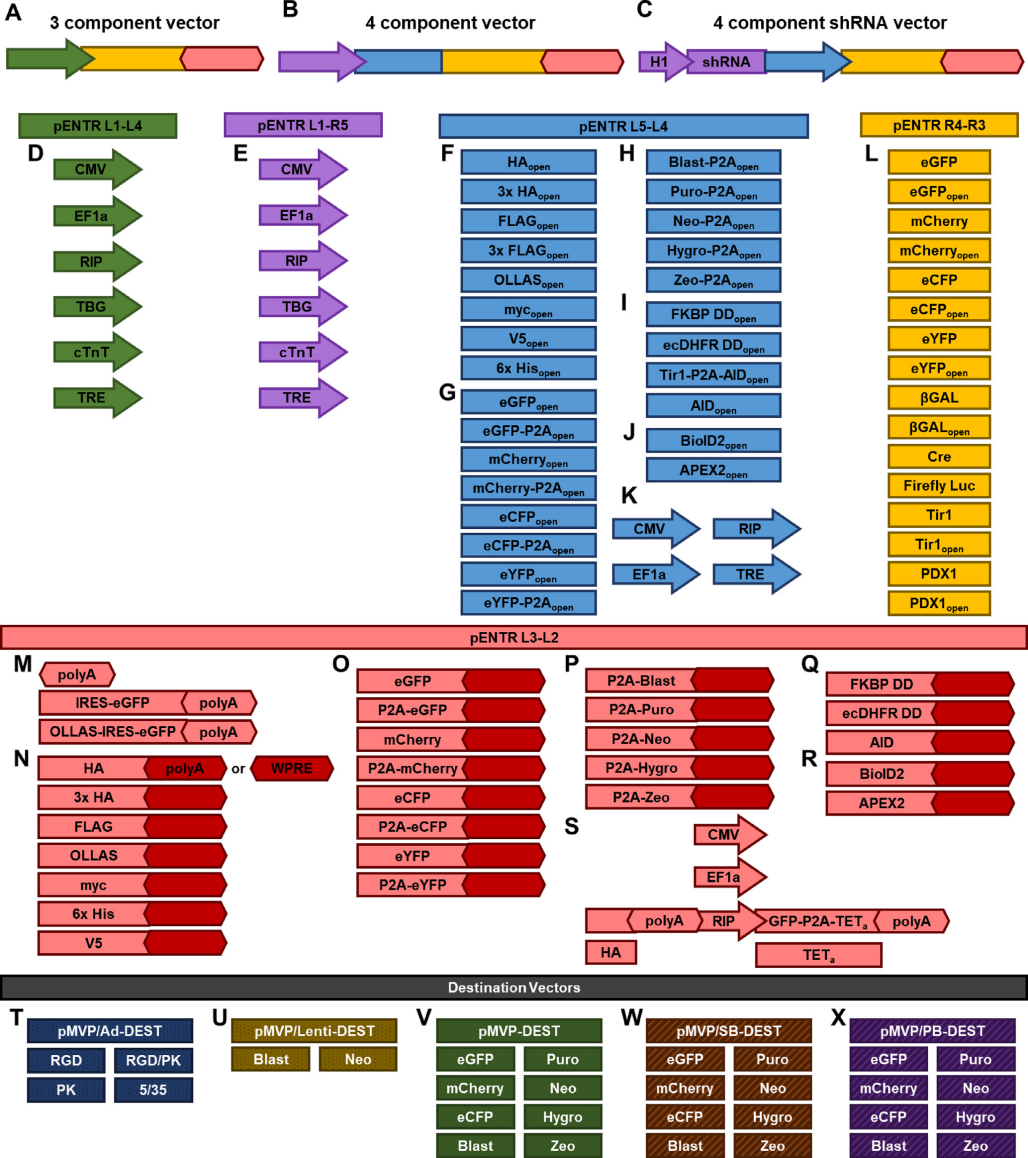
The pMVP toolbox. (**A–C**) A schematic representation of the types of vectors that can be created with pMVP. Color scheme of the components matches the respective vector combinations. Universal pMVP components include: (**D, E, K**) constitutive, cell-type specific, and inducible promoters (arrows); N-terminal (**F**) epitope tags; (**G**) fluorescent protein fusions and P2A reporters; (**H**) mammalian selection markers; (**I**) degron-mediated systems for inducible transgene regulation; (**J**) tools for protein interaction partner identification; and (**L**) an assortment of control genes and genes of interest. (**M**) For polyA permissive vectors, pMVP contains IRES-eGFP reporters. In addition, pMVP also includes components to allow C-terminal fusion of: (**N**) epitope tags; (**O**) fluorescent proteins; (**P**) mammalian selection markers; (**Q**) degron domains; and (**R**) protein identification technologies in combination with either a polyA or WPRE (for lentivirus vectors). (**S**) pMVP also includes a Tet-On system for inducible gene expression, with choice of promoter, eGFP reporter, and fusion of a C-terminal HA tag to genes of interest. Finally, pMVP contains 35 custom options for (**T**) adenoviral, (**U**) lentiviral, (**V**) expression plasmid, (**W**) Sleeping Beauty transposon, and (**X**) PiggyBac transposon destination vectors.

To enable rapid, high-fidelity assembly of unique vectors in a user-friendly manner, we utilized Multisite Gateway® Pro technology (12) as a foundation to generate a suite of entry plasmids (pENTR) and promoterless Gateway destination vectors (pDEST) that we term globally as ‘pMVP’, through which a gene of interest can be paired with combinations of promoter/polyadenylation signal (polyA) (Figures 1A, 2A, 3A) or promoter/N-terminal fusion/polyA (Figures 1A, 2B and 3B) elements and recombined into a pDEST vector (Figures 1B, 2A and B) via an overnight recombination reaction. Thus, in as little as five days, a pENTR plasmid for a new gene of interest can be created and subsequently recombined with pMVP pENTR plasmids to form a unique vector suited for a specific experimental need (Figure 2C). Furthermore, once a gene is added to the system, the cross-vector compatibility of pMVP permits additional unique vectors to be generated within three days with <2 h of benchwork (Figures 1A–C and 2C). Within this framework, we created a toolbox of >100 pENTR MultiSite Gateway Pro building blocks and 35 custom Gateway destination vectors (Figure 3, Supplemental Tables S1 and S3), enabling the creation of >108,000 unique vectors for a gene of interest. To drive gene expression, we incorporated various ubiquitous (CMV, EF1a) and cell-type specific (RIP (8), TBG (23), cTnT (41)) promoters, as well as our recently-published self-contained Tet-On inducible expression system (8), where the Tet-on activator is driven by CMV, EF1a, or RIP (Figure 3D, E, K, S). For transgene detection, an assortment of epitope tags (HA, FLAG, OLLAS, V5, myc, 6x-His) are provided for fusion to either the N-terminus or C-terminus of the recombinant protein of interest (Figure 3F, N). Additionally, fluorescent reporters (eGFP, mCherry, eYFP, eCFP) can be fused to either terminus of a gene or linked as a separate reporter via a P2A ‘self-cleaving’ linker (51) (Figure 3G, O). An eGFP reporter can also be expressed via an IRES sequence (Figure 3M). Similar to fluorescent reporters, mammalian selection markers can also be linked to either terminus of a gene through P2A linkers to create bicistronic vectors (Figure 3H, P). Transgene protein dynamics can be modulated by fusion of the TMP/ecDHFR degron domain (52), Shld1/FKBP degron domain (53), or auxin-induced degron (AID) (40,54) systems (Figure 3I, Q). As described below, these elements allow controlled accumulation or degradation of a target protein by addition of exogenous factors. In addition, protein-protein interaction dynamics for a gene of interest can be examined in living cells through N- or C-terminal fusion of the BioID2 (55) and APEX2 (39) interaction partner identification tools (Figure 3J, R). To accommodate lentivirus vectors, we replaced the polyadenylation signal with a WPRE element for most of the C-terminal fusions (Figure 3N–R). pMVP also contains several control genes as well as genes that have general research utility (Figure 3L). Finally, we generated a pENTR L1-R5 plasmid containing our previously published shRNA expression cassette (3). As an alternative approach for RNAi incorporation, we also designed a shRNA cassette that can be synthesized in a recombination-ready format, precluding the necessity of entry plasmid formation before the LR recombination (Supplemental Figure S1A, B). These shRNA cassettes permit the creation of dual gene vectors capable of simultaneous shRNA and transgene expression (Figure 3C), as is demonstrated in Supplemental Figure S1C–E. This feature of the platform replaces technically challenging dual vector experimental approaches that are currently in use (5).

Current modular systems employ cloning methodologies that are constrained by the requirement for unique restriction sites to linearize the vector before use (56,57). This requirement imposes artificial barriers to the deployment of new technologies, especially in those models reliant on large vectors such as Ad5 that contain limited unique restriction site options. Through the use of λ integrase to mediate restriction-free assembly (12), MultiSite Gateway Pro cloning enables broad cross-vector compatibility that we sought to harness in the pMVP platform. A potential limitation to this goal is the paucity of promoterless pDEST vector options. To overcome this obstacle, we constructed a set of 35 pMVP-compatible, custom pDEST vectors to enable transient (adenovirus, expression plasmid) and stable (lentivirus, SB transposon, PB transposon) transgene expression (Figure 3T–X, Table 1, Supplemental Table S3).

**Table 1.** Comparison of the properties of pMVP vectors. Pertinent information for every pMVP vector family is listed, including vector basis, safety profile, integration bias, and how they are introduced into the cell. Viral vector packaging efficiency is adversely impacted by recombinant vector genomes that greatly exceed the wild-type virus genome. Therefore, the range of insert capacities for all available Ad5 and lentivirus vectors are listed. Insert capacity information for each individual viral vector is listed in Supplemental Table S3. The bottom row includes flow cytometry analysis of eGFP expression from the stable cell lines generated using lentivirus, SB, and PB vectors in Figure 1. After blasticidin selection, the selection agent was removed and the cells were cultured for an additional 3 weeks before analysis

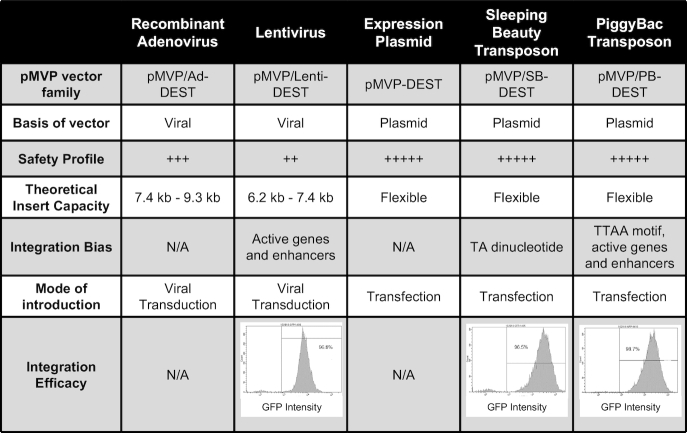

As a starting point for our Ad5 vectors, we engineered pAd/PL-DEST, the commercially available Gateway Ad5 vector, to expand its capacity to accommodate inserts of up to 8.4 kb (pMVP/Ad-DEST). This was accomplished by deleting ∼750 bp of extraneous Ad5 E3 sequence from pAd/PL-DEST to mirror the E3 deletion found in the pAdEasy-1 vector (58). Of note, the deleted E3 sequences were replaced with a unique SgrDI restriction site, thus creating a straightforward scheme for insertion of transgenes and other elements into the former E3 region (Supplemental Figure S2A). Since the downstream LR recombination reaction mediating formation of pMVP-based adenoviruses occurs *in vitro*, an adapted Ad5 backbone can be utilized immediately after isolation to create an adenovirus. In contrast, the widely used AdEasy platform relies on homologous recombination within *Escherichia coli*, requiring the use of specialized electrocompentent *E. coli* strains pre-transformed with the adapted Ad5 genome for adenovirus production (58). Importantly, we can readily achieve purified adenovirus yields >2 × 10^12^ viral particles/ml with pMVP/Ad-DEST, in line with the yields from the unmodified parental vector (data not shown).

One shortcoming of Ad5 vectors is their reliance on the coxsackievirus and adenovirus receptor (CAR), which limits their ability to transduce cell types with low or absent CAR expression. Other groups have identified modifications of the knob region of the Ad5 fiber and chimeric fiber proteins that facilitate CAR-independent transduction (Supplemental Figure S3A) (59–63). To expand the utility of pMVP to cell types resistant to transduction with Ad5, such as C2C12 myoblasts and 3T3-L1 cells, we included fiber-modified adenoviruses in our vector repertoire. To this end, we developed a new pipeline which allows for fiber gene modification and subsequent reinsertion back into our expanded-capacity adenoviral vector (Supplemental Figure S2B). In brief, NEBuilder HiFi DNA Assembly was used to subclone the 1.8 kb BarI/BstBI Ad5 fiber gene fragment and two linker gBlocks (JH512/JH513) into a KpnI/SacI digested pBlueScript2SK(–) shuttle plasmid, simplifying the cloning process for any subsequent fiber modifications. The resulting shuttle plasmid, pBS/Fiber, was engineered to contain restriction sites for the Type IIS enzyme BsmBI, so that upon digestion, the modified fiber gene fragment is released with sufficient flanking Ad5 sequence for scarless reassembly into a BarI/BstBI digested pMVP/Ad-DEST vector. This approach was used to generate adenovirus vectors containing the RGD (59,63), pK (59) or RGD/pK (61) fiber modifications in addition to a chimeric Ad5/35 (60,62) fiber gene (Figure 3T). As expected, expression of eGFP from an adenovirus containing a RGD fiber modification dramatically enhanced transduction of C2C12 myoblast cells when compared to a wild-type Ad5 vector (Supplemental Figure S3B and C).

Many expression vectors contain unnecessary components that increase overall vector size and can lead to decreased transfection efficiency. To engineer a compact, pMVP-compatible expression vector, we inserted a Gateway cloning cassette into pBlueScript2SK(-) (pMVP_BS_-DEST) and subsequently removed all non-essential plasmid components to generate pMVP-DEST. We then introduced selection markers (blasticidin, puromycin, neomycin, hygromycin, zeocin) or fluorescent reporters (eGFP, mCherry, eCFP) to facilitate multiple use modalities (Figure 3V). Using pMVP-DEST as a backbone, we created SB (pMVP/SB-DEST) and PB (pMVP/PB-DEST) transposon destination vectors. The SB and PB transposon systems are synthetic DNA transposons that enable highly efficient and stable incorporation of foreign DNA into a host genome dictated by the presence of a TA dinucleotide (SB) or TTAA tetranucleotide (PB) (35,42,64). Critically, transposons faithfully transfer the entirety of the gene expression cassette into the host genome, unlike plasmid integration techniques, while also avoiding the biosafety concerns associated with lentiviruses. Of note, SB-mediated integration is agnostic to chromatin state, thus diminishing the chance that an essential locus will be disrupted. In contrast, both PB- and lentiviral-mediated integration have been observed to exhibit a strong bias towards active genes and enhancers (65,66) (Table 1). We made SB and PB destination vectors for pMVP by inserting the respective transposon ITR elements on either side of the pMVP-DEST Gateway cloning cassette. We then inserted the selection markers and fluorescent reporters listed above between the attL2 site and adjacent ITR to enable selection of transformed cells (Figure 3W–X).

Despite the drawbacks discussed above, lentivirus vectors remain a highly utilized tool due to their ability to efficiently transduce a broad range of cells. Therefore, we generated a pMVP-compatible third-generation lentivirus vector conferring resistance to blasticidin by deletion of the CMV promoter and WPRE element from pLenti6.1/V5-DEST. We also replaced the blasticidin resistance gene with a neomycin resistance gene and deleted the eukaryotic selection cassette (Figure 3U). Finally, we note that pMVP can be readily adapted to include alternative destination vectors, promoters, epitope tags, reporters, or other technologies by strategies similar to those outlined here.

### Demonstration of pMVP utility

To demonstrate the broad flexibility and unique cross-vector compatibility of pMVP, we inserted the cDNA encoding the human homeobox transcription factor PDX1, with or without a stop codon (i.e. open), into the platform (Figure 3L) via PCR. We then subsequently recombined PDX1_open_ with pMVP plasmids containing the EF1a promoter, eGFP-P2A, and 3× HA epitope tag/polyA (or WPRE) into one member of the five pDEST vector subtypes (Ad5, lentivirus, expression, SB, PB) (Figure 1). As shown in Figure 1D, immunoblot analysis confirmed successful construction of each of these vectors and corresponding viruses. These vectors enabled overexpression of an eGFP reporter and PDX1-3x HA tagged fusion protein within the contexts of primary rat islets (Ad5), stable cell lines (lentivirus, SB, PB), and transient transfection of HEK293 cells (expression). When subjected to flow cytometric analysis, the purity of the resulting stable cell lines from lentivirus, SB, and PB transformation were found to be > 96%, indicative of both efficient selection and stable transgene expression (Table 1).

**Figure 4.**
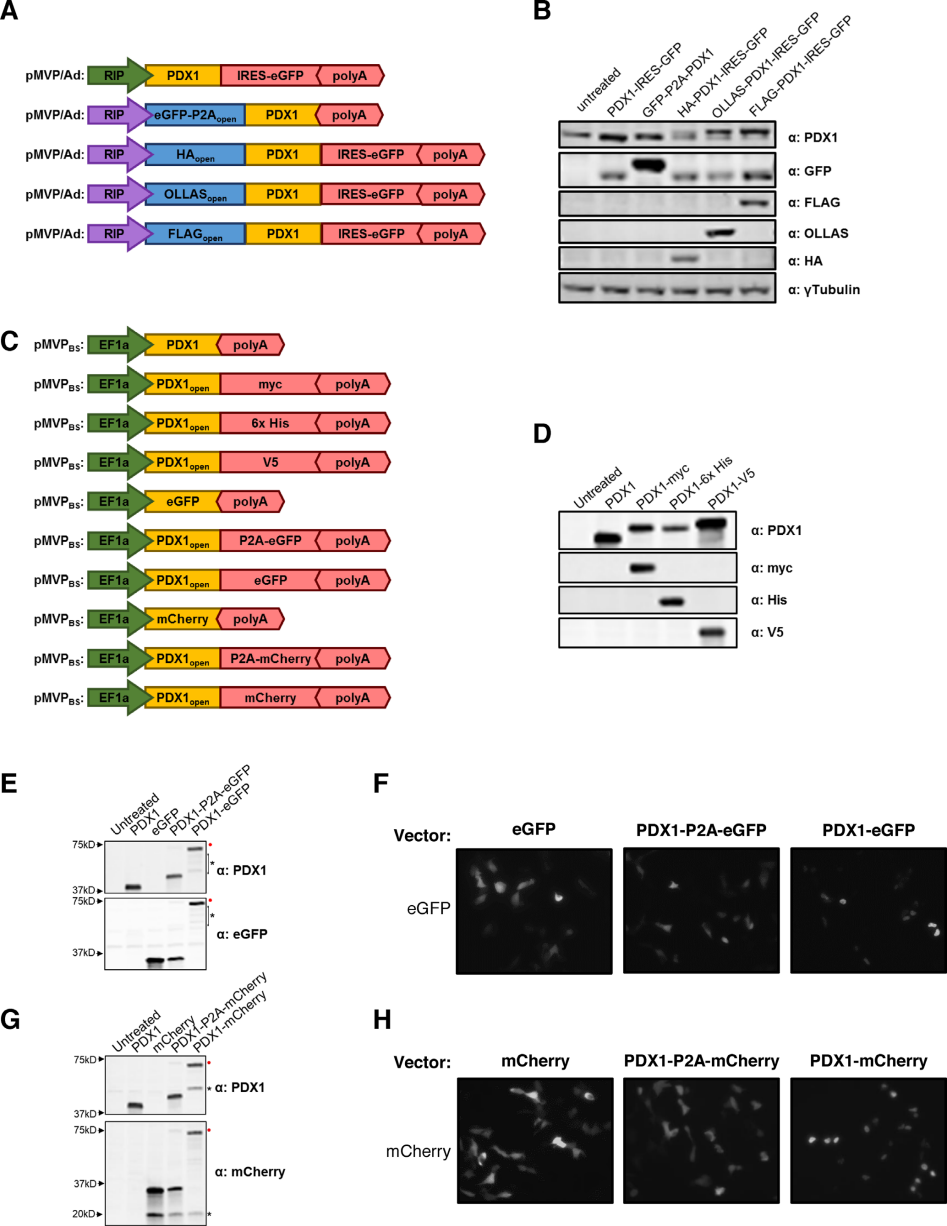
Utilization of pMVP for the creation of unique PDX1 vectors. pENTR plasmids containing cDNA for human PDX1 with or without a stop codon (i.e. open) were recombined with pMVP components to generate an assortment of PDX1-expressing (**A**) adenovirus and (**C**) expression plasmid vectors. (**B**) Immunoblot blot analysis of INS1 832/13 cell lysates harvested 48h after treatment with crude adenovirus lysates. For the epitope tagged conditions, note the appearance of the endogenous (lower) and overexpressed (upper) PDX1 bands (top blot). For eGFP blot, the use of P2A adds 23 amino acids to the N-terminal protein (i.e. eGFP). Immunoblot analysis of HEK293 cell lysates 24 h after transfection of expression vectors encoding PDX1 with C-terminal (**D**) epitope tags, (**E**) eGFP reporter or fusion, (**G**) and mCherry reporter or fusion. (**F, H**) Fluorescence microscopy of the eGFP and mCherry containing conditions analyzed in panels E and G. Visible bands resulting from ‘Uncleaved’ P2A products (red circle •) and degradation products (*) are labeled.

To validate the ability of pMVP to create experimentally useful fusion proteins, we generated 13 unique PDX1 Ad5 or expression plasmid vectors containing N-terminal epitope tags and/or eGFP reporters, or C-terminal eGFP/mCherry/epitope tag fusions or P2A-based bicistronic vectors with fluorescent reporters (Figure 4A, C). Immunoblot analysis confirmed overexpression of the desired PDX1 variants upon adenoviral transduction of INS1 832/13 cells (Figure 4B) or expression plasmid transfection into HEK293 cells (Figure 4D, E, G). These analyses also demonstrate that nearly all of the protein emanating from the P2A-linked bicistronic vectors was ‘cleaved’, although a faint band representing the ‘uncleaved’ fusion protein was visible (Figure 4E, G, red circle), as has been previously reported (51). Furthermore, using fluorescent microscopy we confirmed that P2A-linked eGFP and mCherry reporters remained diffuse throughout the cell indicative of successful ‘cleavage’. In contrast, when fused to PDX1 these fluorescent proteins accumulated in the nucleus as expected (Figure 4F, H).

**Figure 5.**
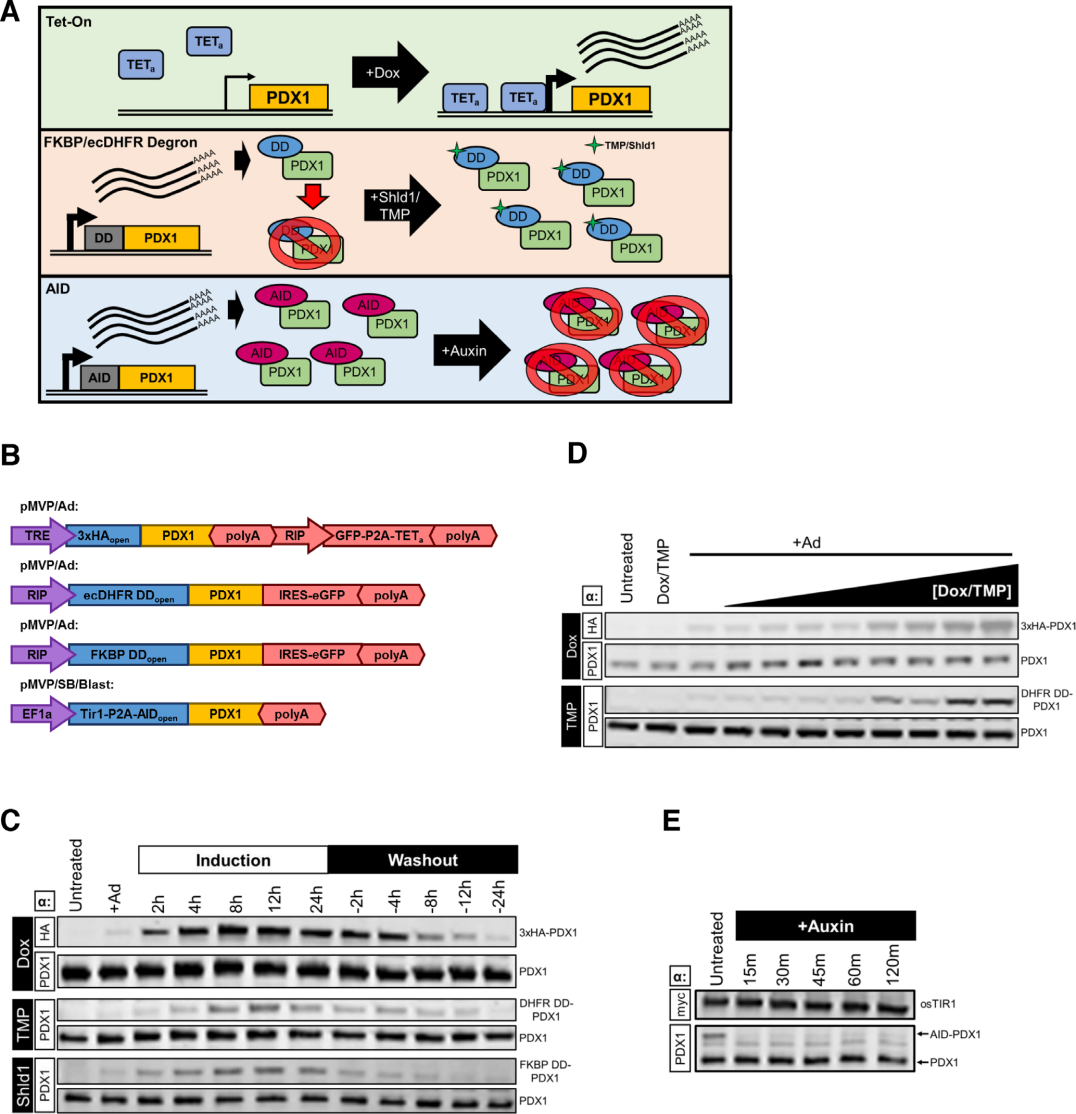
Demonstration of pMVP inducible systems. (**A**) Graphical depiction of the mechanisms for the Tet-On, FKBP/ecDHFR degron domain, and AID systems and (**B**) the pMVP vectors generated and utilized. (**C**) Immunoblot analysis of INS1 832/13 cell lysates transduced with crude lysates for adenoviruses expressing PDX1 using either the Tet-On, ecDHFR, or FKBP systems and harvested at the indicated time intervals after addition (induction) and removal (washout) of the respective Dox, TMP, or Shld1 inducers. **(D)** Immunoblot analysis of INS1 832/13 cell lysates transduced with crude lysates for adenoviruses expressing PDX1 using either the Tet-On or ecDHFR systems and harvested 24h after addition of a dose curve of Dox (0.5–100 ng/ul) or TMP (1 nM–5 uM), respectively. (**E**) Immunoblot analysis of cell lysates from an INS1 832/13-derived cell line made by Sleeping Beauty-mediated integration of Tir1-P2A-AID-PDX1. Lysates were harvested at the indicated time intervals after treatment with 500uM auxin. (C–E) Endogenous PDX1 and PDX1-fusion bands were distinguished based upon size or the use of an epitope tag and are labeled accordingly.

Temporal control of transgene expression is a highly utile experimental tool, but not readily available for use in many vectors. We therefore incorporated Tet-On, TMP and SHLD1 degron domain stabilization, and AID technologies (see overview in Figure 5A) for temporal transgene control in pMVP. The widely implemented Tet-On system utilizes a constitutive promoter to drive expression of the Tet-On transactivator (TET_a_), which upon doxycycline (Dox) addition, binds to and activates expression from TRE (Tet-response element) promoters (8). Conversely, degron domain technology requires fusion of a synthetic degron domain derived from either FKBP12 (108 a.a.) or ecDHFR (159 a.a.) to cause rapid protein degradation. Addition of a small molecule ligand (Shld1 or TMP respectively) stabilizes the degron domain and induces transgene protein accumulation (52,53). The AID system requires fusion of a 68 amino acid minimal AID tag (54) in addition to constitutive overexpression of an auxin perceptive F-box plant protein, osTir1 that can interact with the mammalian SCF complex. Under basal conditions, AID-tagged transgenes are overexpressed; addition of the plant phytohormone auxin triggers rapid transgene degradation through interaction with osTir1 (40,54). In order to streamline utilization of the AID system, we utilized a P2A-linker to enable creation of bicistronic vectors that simultaneously express osTIR1 and an AID-tagged gene of interest (Figure 3I). To demonstrate the utility of these tools, we generated adenoviruses that directed inducible PDX1 expression in response to Dox, TMP or Shld1. We also generated a SB vector to create an INS1 832/13-derived cell line stably expressing osTir1-P2A-AID-PDX1 (Figure 5B). As expected, INS1 832/13 cells transduced with the Ad5 constructs induced expression of PDX1 upon addition of Dox, TMP or Shld1, and this expression was reversed upon removal of the respective inducers (Figure 5C). Additionally, the level of transgene expression could be controlled by varying the dose of inducer added to the system (Figure 5D). Finally, we evaluated the dynamics of the AID system through addition of auxin to our AID-PDX1 expressing cells. Remarkably, overexpressed human PDX1 protein was almost completely degraded within 15 minutes of auxin addition, whereas endogenous rat PDX1 levels remained unchanged (Figure 5E). In summary, upon insertion of PDX1 into pMVP, we were able to rapidly create 22 unique vectors to allow PDX1 expression in the context of a range of epitope tags, eGFP reporters and various modalities for inducible expression. Again, it should be noted that these 22 unique vectors represent only a small fraction of the >108,000 possible PDX1 vector combinations, not including RNAi, that are possible with the pMVP system.

### Creation of the epigenetic engineering platform pMAGIC

**Figure 6.**
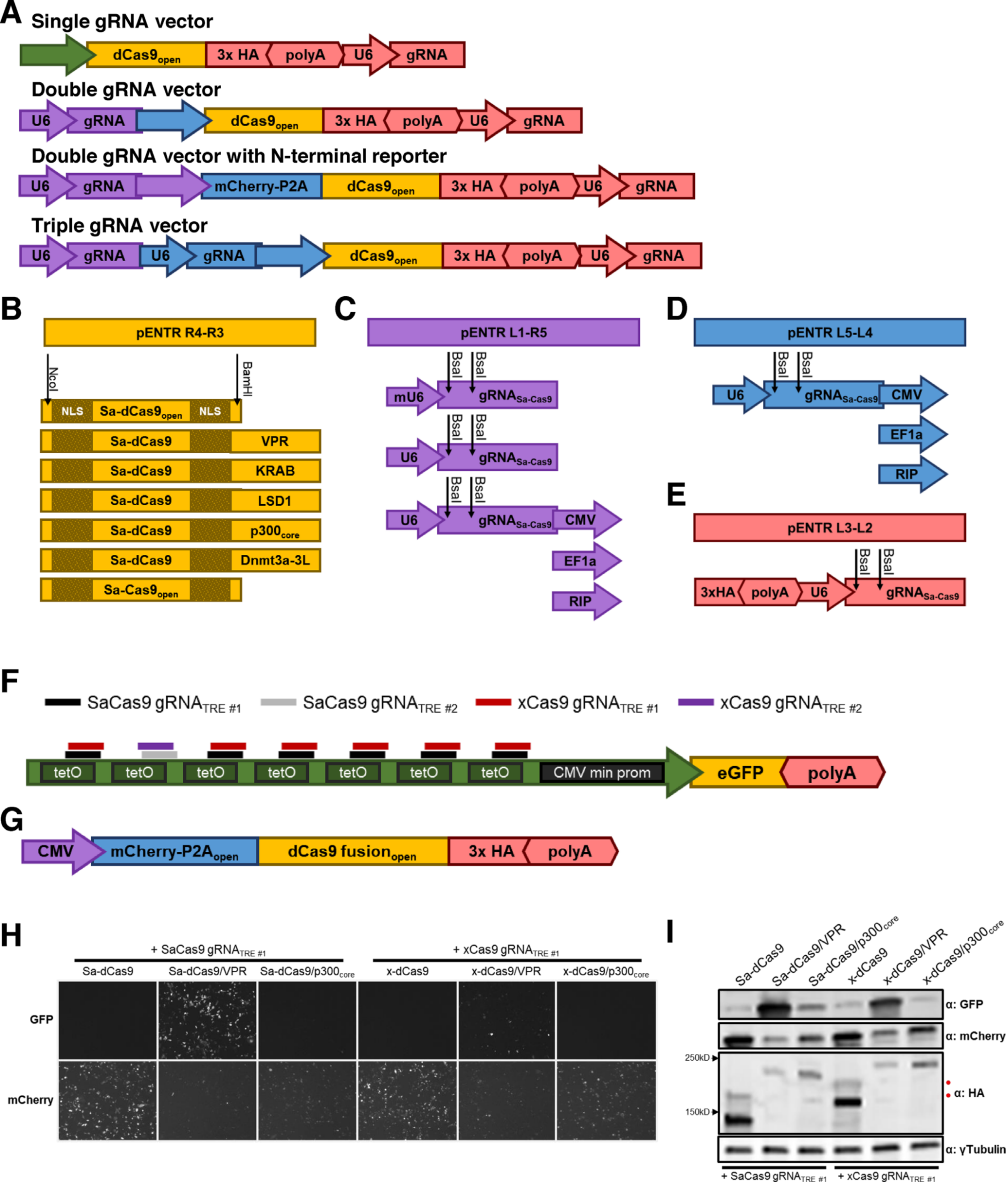
The pMAGIC toolbox. (**A**) A schematic representation of the structure of pMAGIC-derived single, double, and triple gRNA expressing vectors. Color scheme of the components matches the respective vector combinations. (**B**) Sa-dCas9 and Sa-Cas9 pMAGIC fusion genes cloned into pENTR221 R4-R3 (yellow). Additional effectors can be fused to Sa-dCas9 through unique NcoI (N-terminal) and BamHI (C-terminus) restriction sites. (**C**) pENTR221 L1-R5 plasmids (purple) containing a Sa-Cas9 gRNA expression cassette driven by the mouse U6 (mU6) or human U6 (U6) promoter. Alternatively, a U6-driven Sa-Cas9 gRNA expression cassette can be combined with a CMV, EF1a, or RIP promoter. (**D**) pENTR221 L5-L4 plasmids (blue) containing a U6-driven Sa-Cas9 gRNA expression cassette and CMV, EF1a, or RIP promoter. (**E**) pENTR221 L3-L2 plasmid (red) containing a 3xHA epitope tag for C-terminal fusion to Sa-Cas9 proteins and a U6-driven Sa-Cas9 gRNA expression cassette. (C–E) Protospacer oligonucleotides can be ligated into BsaI digested gRNA expression cassettes. (**F**) Sa-Cas9 and x-Cas9 gRNA binding sites within the TRE promoter. Schematic of the TRE-driven eGFP reporter used to monitor dCas9-mediated transcriptional activation. (**G**) Schematic of mCherry-P2A-dCas9 expression vectors. HEK293 cells were co-transfected with plasmids for the eGFP reporter shown in panel (F), equimolar amounts of the indicated mCherry-P2A-dCas9 vectors, and the corresponding Sa-Cas9 gRNA_TRE#1_ or x-Cas9 gRNA_TRE #1_ plasmids. 72h after transfection, cells were (**H**) visualized by fluorescence microscopy and (**I**) cellular lysates were analyzed by immunoblot. ‘Uncleaved’ P2A products from Sa-dCas9 and x-dCas9 are designated by a red circle (•).

dCas9-mediated epigenetic engineering has the potential to transform our understanding of the genetic underpinnings of disease. However, the ability to apply these tools in primary cells relevant to disease, such as the pancreatic islet, is limited by the lack of vector options. In addition, the large size of dCas9 fusions cannot be accommodated in many vector contexts. We felt that these restrictions could be resolved through the use of Ad5 vectors. To test this, we employed the pMVP framework to develop a modular epigenetic engineering platform, pMAGIC, that allows a promoter of choice, Sa-dCas9 or x-dCas9(3.7) fused to one of five epigenetic modifier proteins, and up to three (Sa-dCas9) or two (x-dCas9(3.7)) gRNAs, to be recombined into any of the pMVP adenovirus vectors (Figure 6A). Of note, pMAGIC components are also compatible with other pMVP vectors, provided the vector has sufficient insert capacity to accommodate and efficiently deliver the desired components (see Table 1 and Supplemental Table S3 for vector overview and insert limitations).

While the vast majority of epigenetic engineering applications to date have utilized Sp-dCas9 (∼4 kb), our initial efforts utilized the Sa-dCas9 ortholog (∼3 kb) (23) that, to our knowledge, has only been previously used for transcriptional modulation (37) and not epigenetic engineering. The utilization of Sa-dCas9 enables the packaging of a RIP-driven Sa-dCas9/LSD1 fusion, one gRNA, and polyA (total size ∼7.1 kb) into the standard Gateway Ad5 vector (pAd/PL-DEST, maximum insert ∼7.6 kb). Alternatively, the extra ∼750 bps available in our expanded capacity Gateway Ad5 (pMVP/Ad-DEST, maximum insert ∼8.4 kb) enables a total of 3 gRNAs to be packaged alongside Sa-dCas9/LSD1 (total size ∼7.8 kb). Using PCR, we generated ‘gene of interest’ entry plasmids for either humanized Sa-dCas9 flanked by nuclear localization sequences (NLS) or Sa-dCas9 fused to VPR, a tripartite transcriptional activator composed of VP64-p65-Rta (17,37) (Figure 6B). Subsequently, we utilized the unique BamHI site downstream of Sa-dCas9 to fuse with one of five effectors: KRAB, the Krűppel associated box transcriptional repressor domain which recruits H3K9 methyltransferases (16,20); p300_core_, the H3K27 acetyltransferase domain of p300 (18); LSD1, a H3K4me1/2 demethylase (19,21); or Dnmt3a-3L, a synthetic DNA methyltransferase formed by fusion of the C-terminal domains of Dnmt3a and Dnmt3L (22). Note that alternative effectors can be readily added to the system via the unique NcoI and BamHI restriction sites flanking Sa-dCas9 (Figure 6B). To enable multiplexed gRNA expression, we constructed entry plasmids containing solely the human U6 promoter and a Sa-Cas9 gRNA sequence modified to enhance expression (46) (Figure 6C), or the gRNA expression cassette in combination with either a promoter (Figure 6C, D) or 3xHA bGH polyA (Figure 6E). Protospacer oligonucleotides corresponding to a specific target sequence can be ligated into any BsaI digested pENTR plasmid containing the Sa-Cas9 gRNA expression cassette (Figure 6C–E, Supplemental Figure S4D). Importantly, the modularity of pMAGIC allows gRNA expression plasmids to be mixed and matched with each other, as well as the various Sa-dCas9-effectors, thereby enabling the creation of complex vectors targeting up to three distinct genomic loci with a targeted epigenetic modifier (Figure 6A). To diminish the potential for unwanted recombination events due to the repetitiveness of packing three gRNAs, we designed the gRNAs in a head-to-tail formation that is more stable in *E. coli* than the head-to-head formation used by others (67) (Supplemental Figure S4H). However, as an alternative we have also included a mU6-driven gRNA cassette that can also be utilized if preferred (Figure 6C, Supplemental Figure S4F). While not pursued here, it is worth noting that additional gRNA expression cassettes can also be inserted into the unique SgrDI restriction site we engineered in the Ad5 E3 region (Supplemental Figure S2B). Furthermore, since pMVP is the foundation of pMAGIC, gRNA expression cassettes can be replaced with any of the epitope tags, fluorescent reporters, transgene regulatory technologies, or protein identification tools, thus, adding another layer of flexibility to design of dCas9-based experiments.

Recently, a Sp-Cas9 mutant that exhibits exceptional PAM flexibility (NG, NNG, GAT, CAA), referred to as x-Cas9(3.7), has been described (24). Despite the limitations imposed by the larger size of x-Cas9(3.7), the exponential expansion of potential genome target sites warranted inclusion in pMAGIC. As such, we made a suite of x-dCas9(3.7) fusions that mirror our Sa-dCas9 options (Supplemental Figure S4A–C, E, G). Inclusion of the larger x-dCas9(3.7) construct limits the number of potential multiplexed gRNAs to two. Of note, despite the 6.8 kb size of the x-dCas9(3.7)/LSD1 gene being restrictive for efficient production of lentivirus (68) and pAd/PL-DEST vectors, the expanded packaging limit of our new pMVP/Ad5 vectors can theoretically accommodate a RIP-driven construct with up to 2 gRNAs (total size ∼ 8.3 kb). As is evidenced by the invention of xCas9(3.7), the Cas9 field is rapidly evolving. To enable future Cas9 discoveries to be easily applied to the epigenetic engineering field, we developed a series of pENTR plasmids that enabled x-dCas9(3.7), or any future Cas9-variant, to be inserted between two NLS sequences and fused to the effectors described here (Supplemental Table S2). Additionally, since the components and orthogonal Cas9 genes are now housed in the same molecular framework, pMAGIC lays the foundation for comparative studies to gain a better understanding of the nuances that exist between the various dCas9 orthologs.

To test dCas9-mediated targeting of effectors to a specific DNA sequence, we generated pMAGIC expression vectors for constitutive expression of a mCherry-P2A reporter and 3xHA epitope-tagged Sa-dCas9 or x-dCas9(3.7) alone, or in combination with the VPR or p300_core_ effector domains (Figure 6G). As a reporter of transcriptional activation, we used pMVP to generate a plasmid containing eGFP expressed from the inducible TRE promoter. The repetitive nature of the ∼300 bp TRE promoter enabled us to design gRNAs that target the promoter either six (gRNA_TRE #1_) or a single (gRNA_TRE #2_) time for both Sa- and x-Cas9(3.7) (Figure 6F). Equimolar amounts of each mCherry-P2A-dCas9 vector were then co-transfected into HEK293 cells in combination with the eGFP reporter vector and a corresponding gRNA plasmid for analysis by fluorescence microscopy and immunoblot. Within this context, both Sa-dCas9/VPR and x-dCas9(3.7)/VPR strongly induced eGFP expression when targeted six times to the TRE with gRNA_TRE #1_ (Figure 6H, I). As previously reported (69), p300_core_ fusions had either modest (Sa-dCas9) or negligible (x-dCas9(3.7)) activation relative to VPR fusions (Figure 6H, I). Targeting the TRE only once using gRNA_TRE #2_ produced similar trends (Supplemental Figure S4I, J). Unexpectedly, we also observed dramatic expression differences for both mCherry and HA-tagged dCas9 that were correlated with the dCas9 effector (Figure 6H–I, Supplemental Figure S4I, J). As the expression of mCherry was also impacted, these observations are indicative of significant differences in either the transcription or translation rates of the various fusions dictated by the fusion partner rather than a change in protein half-life. Importantly, these data highlight the importance of using ‘in-kind’ controls for dCas9 experiments whenever possible, as the large differences in expression level we observed have the potential to confound interpretation of downstream analyses. In total, we have successfully created a highly versatile, modular epigenetic engineering platform, pMAGIC, that enables the locus-specific recruitment of a variety of effectors and is compatible with pMVP, allowing for tailoring of vectors to achieve experimental goals.

### Targeting Sa-dCas9/LSD1 to PDX1 Area IV is sufficient to repress PDX1 expression and alter PDX1 target genes

pMAGIC was designed to permit epigenetic engineering studies in difficult model systems, including pancreatic islets. Furthermore, the compatibility with Ad5 vectors also allows efficient expression of the large dCas9/LSD1 fusion in a broad array of cell types; previously, dCas9/LSD fusions had only been studied in a single stable cell line (19). Importantly, LSD1 has been suggested to be the only epigenetic modifier in the dCas9 toolbox with a selective bias for repressing enhancers versus promoters (19). Thus, pMAGIC allows efficient delivery of this impactful and specific tool in a much broader array of biological contexts.

**Figure 7.**
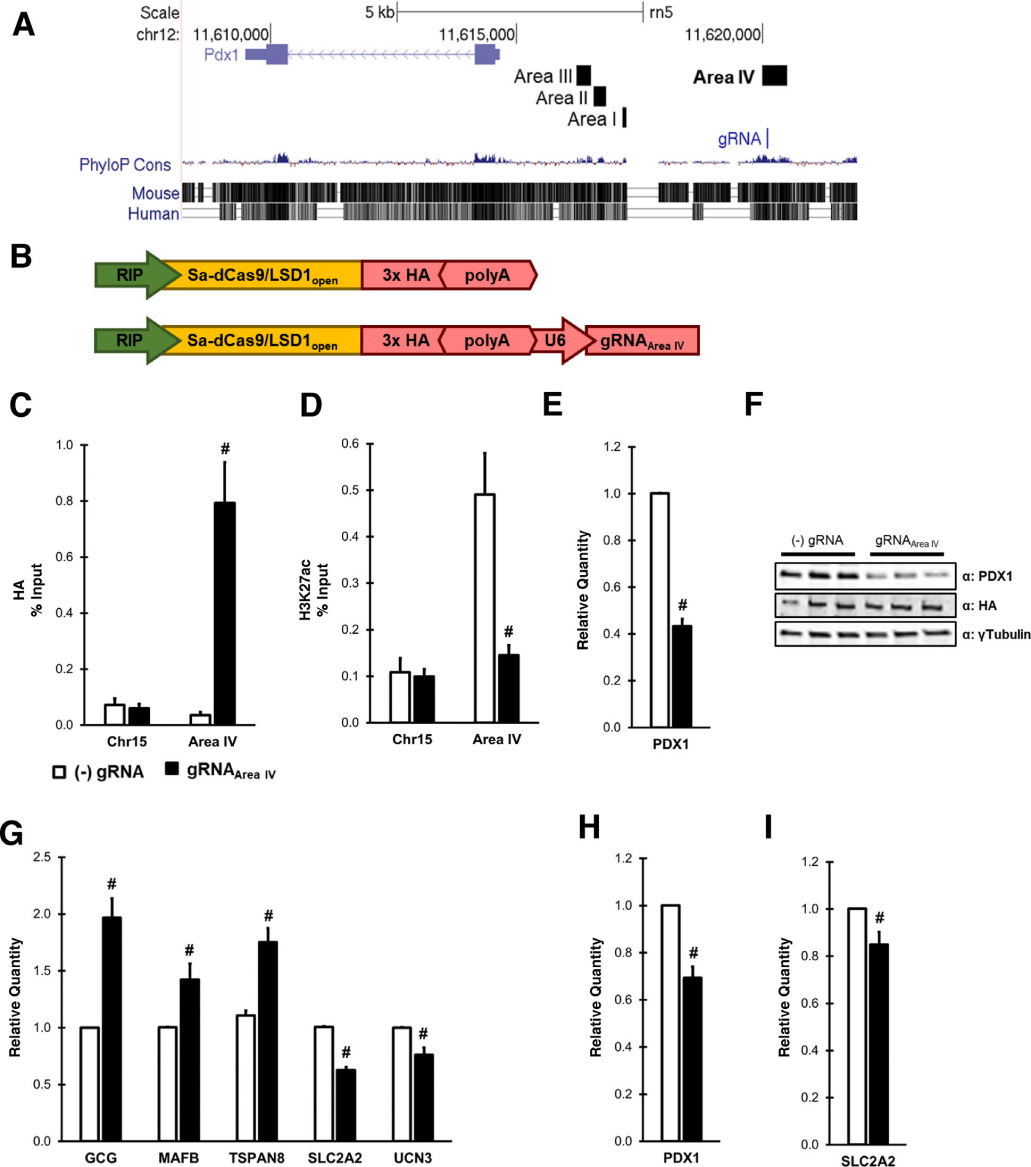
Targeting Sa-dCas9/LSD1 to PDX1 Area IV is sufficient to decrease PDX1 and disrupt PDX1-target gene expression. (**A**) UCSC genome browser screenshot of the PDX1 locus in the rat genome (rn5). Highlighted areas demark Area I-IV and gRNA_Area IV_ targeting within Area IV. (**B**) Schematic of pMAGIC-derived adenoviruses expressing Sa-dCas9/LSD1. ChIP-qPCR analysis of (**C**) HA epitope tag and (**D**) H3K27ac enrichment using primers to a control region (Chr15) or Area IV in INS1 832/13 cells. Data are shown as a percent of input. (**E**) qRT-PCR analysis of PDX1 expression levels in INS1 832/13 cells. (**F**) Immunoblot analysis of INS1 832/13 cell lysates from 3 independent experiments. (**G**) qRT-PCR gene expression analysis in INS1 832/13 cells for genes directly regulated by PDX1. qRT-PCR analysis of (**H**) PDX1 and (**I**) the PDX1 target gene SLC2A2 (GLUT2) in intact primary rat islets. For all experiments, cells were harvested 48 h after transduction with either (–) gRNA control (white) or gRNA_Area IV_ (black) purified adenoviruses depicted in panel B. Data represent mean ± S.E.M. of 3 (E, G) or 4 (C, D, H, I) independent experiments. ^#^*P* < 0.05 compared to (–) gRNA control adenovirus.

To demonstrate this, we focused on a highly conserved 0.5 kb enhancer element, referred to as Area IV, located approximately 5.5 kb upstream of the PDX1 transcription start site (Figure 7A). Previous work has demonstrated that the neighboring Areas I-III regulate PDX1 expression during development, whereas Area IV only becomes active during β-cell maturation (25). A recent genome-wide association study has identified fasting glucose-associated SNPs in a 40 kb region encompassing Areas I-IV in adult humans, suggesting that dysregulation of PDX1 enhancers may impact glucose homeostasis (33). However, the role of Area IV in maintaining PDX1 expression in mature β-cells has not been elucidated.

To further investigate the effect of direct manipulation of epigenetic status of Area IV, we used pMAGIC to create a RIP-driven, β-cell specific adenoviral vector that targets Sa-dCas9/LSD1 to Area IV using a single gRNA (gRNA_Area IV_); the identical vector lacking a gRNA was used as the control (Figure 7A, B). Transduction of INS1 832/13 cells with these vectors, followed by chromatin immunoprecipitation (ChIP) analysis demonstrated that Sa-dCas9/LSD1 was successfully recruited to Area IV, coinciding with a significant decrease in H3K27ac enrichment, a previously reported marker of both LSD1 and enhancer activity (19,21,70) (Figure 7C, D). To determine whether this perturbation had any functional consequence on PDX1 expression or the PDX1 transcriptional network, we performed qRT-PCR for PDX1 as well as a set of canonical genes previously described to be directly regulated by PDX1 (26,27,71). Remarkably, we observed a robust decrease of >50% in PDX1 mRNA and protein expression (Figure 7E, F). This decrease coincided with significant alterations in the PDX1 direct target genes TSPAN8, SLC2A2 (GLUT2) and UCN3 (Figure 7G). Interestingly, we also observed increases in the PDX1 direct target genes GCG and MAFB that are associated with α-cell identity (26) (Figure 7G). A second gRNA targeting Area IV was also tested, but did not alter PDX1 mRNA expression (data not shown). Currently, the appropriate controls for dCas9-mediated epigenetic engineering are still being debated. To ensure that our observations were indeed due to Sa-dCas9/LSD1 targeting, we utilized the flexibility of pMAGIC to generate several additional control vectors to test whether inclusion of a functional, but non-targeting, gRNA (Sa-Cas9 gRNA_TRE #1_, Figure 6F), or targeting Sa-dCas9 to Area IV modulate PDX1 expression (Supplemental Figure S5A). Additional experiments utilizing these vectors clearly demonstrated that the observed decreases in PDX1 are due to Sa-dCas9/LSD1 targeting to Area IV by gRNA_Area IV_ and not due to conformational shifts that occur upon gRNA/Cas9 interaction, or steric interference of Sa-dCas9 at Area IV (Supplemental Figure S5B–E). Furthermore, in line with our transient transfection experiments in Figure 6, we found striking protein expression level differences between Sa-dCas9 and Sa-dCas9/LSD1 (Supplemental Figure S5E), demonstrating the importance of choosing an ‘in-kind’ control for dCas9 experiments. Taken together, these results implicate epigenetic modification of Area IV as a significant modulatory event for control of PDX1 expression in this rodent insulinoma cell line model.

Lastly, we repeated the experiment using primary rat islets. Confirming our cell line results, PDX1 expression was significantly diminished by 30% upon targeting of PDX1 Area IV with Sa-dCas9/LSD1 (Figure 7H). Furthermore, the PDX1 targeted gene SLC2A2, chosen because its expression is confined to the β-cell, was also significantly decreased (Figure 7I), albeit to a lesser extent than observed in INS1 832/13 cells. Importantly, these experiments represent the first example of LSD1-based epigenetic engineering in a primary tissue. In summary, we used pMAGIC to rapidly assemble an Ad5 vector capable of targeting and modifying a discrete genomic locus in both INS1 832/13 cells and primary rat islets, resulting in a significant decrease of PDX1 expression and perturbation of the PDX1 transcriptional network.

## DISCUSSION

Here, we have described the design, construction, and utilization of innovative modular cloning platforms capable of rapid production of a broad array of unique Ad5, lentiviral, expression plasmid, PB transposon, and SB transposon vectors for the purposes of transgene expression (pMVP, Figure 3) and dCas9-based epigenetic engineering (pMAGIC, Figure 6 and Supplemental Figure S4). In total, these systems offer 192 unique components (Supplemental Tables S1–S3) that provide researchers with broad flexibility to create novel vectors to fulfill a host of experimental demands with markedly less effort than traditional cloning methods. Using these systems, upon incorporation of a single gene such as PDX1 into pMVP, it is possible to rapidly generate in excess of 108,000 unique transgene expression vectors. While pMVP is currently at a disadvantage compared to first generation Gateway approaches that have an abundance of available genes due to the use of the pDONR223 plasmid for the human ORFeome project (72), the options for application of these genes are limited to only a handful of compatible vectors. Furthermore, the flexibility of pMVP will continue to increase exponentially due to the modular framework on which it is built.

Multisite Gateway Pro cloning was chosen as the basis for pMVP due to the distinct advantages over other one-pot cloning methodologies in that it is not limited by size (i.e. CPEC), reliant on unique restriction enzyme sites (i.e. Golden Gate, Gibson assembly), does not utilize error-prone PCR amplified components in the final assembly (i.e. CPEC, Gibson assembly), and is not plagued by imperfect junction assembly (i.e. Gibson assembly) (73–75). Furthermore, the positive (kanamycin/ampicillin) and negative (ccdb) selection built into the Gateway platform (Figure 2A, B) ensures low background colony growth emanating from unrecombined starting reagents. These features enabled us to create a broad library of inserts and destination vectors that are not constrained by the presence/absence of restriction enzyme sites, allowing a ‘plug-and-play’ approach to vector production where plasmids can simply be combined in the recombination reaction without an initial PCR reaction or restriction digest. Whereas others have applied the Multisite Gateway Pro system to generate complex multigene lentivirus vectors (76), such vectors rely on conventional restriction enzyme cloning to create each expression cassette. In contrast, once a gene is inserted into pMVP, the platform solely utilizes Gateway recombination. This design feature means that our system requires minimal molecular biology expertise, which when coupled with the many destination vector options in the platform, provides a highly efficient, user-friendly, and versatile platform for manipulation of gene expression in mammalian cells. Notably, while our current platforms rely on Ad5 and the other vectors described here, additional vectors can readily be added to the system simply through the insertion of a Gateway cloning cassette.

A key feature of pMVP is the broad cross-vector compatibility that, to our knowledge, is unique among modular cloning platforms. This flexibility enables researchers to rapidly develop additional experimental paradigms throughout a project as their hypotheses evolve. The utility of this was highlighted in our recently published work wherein we identified a mechanistic link between branched chain amino acid and lipid metabolism. Using pMVP, we were able to swiftly prepare both expression plasmid and Ad5 vectors expressing a cytoplasmic-restricted mutant form of the branched-chain ketoacid dehydrogenase kinase (BDK), thereby contributing to our discovery of a novel function of this kinase to phosphorylate and regulate the key cytosolic lipogenic enzyme ATP-citrate lyase (77).

Since the first experiment demonstrating the potential of direct manipulation of native genomic elements over 20 years ago using zinc finger proteins (ZFP) (78), the field has been limited by the arduous and low-throughput manner in which ZFPs and the more recently developed transcriptional activator-like effectors (TALE) are generated, although new advances are improving the process (22). The advent of Cas9-based technologies has rapidly brought epigenetic engineering into the mainstream of biomedical research (recently reviewed in (79)). This is due to the unique flexibility of Cas9, which can be retargeted to a new locus simply by inclusion of a ∼20 bp substitution in the gRNA protospacer motif rather than redesign of the entire protein as required for ZFPs and TALEs. However, despite the growing number of effectors within the Cas9 epigenetic engineering toolbox, there remains a lack of uniformity in the manner by which they can be applied. With pMAGIC, these technologies have been assembled into a platform, facilitated by the versatility of the pMVP system. In order to accommodate promoters, multiple gRNAs, and large Cas9 effectors in our vectors, pMAGIC incorporates Sa-Cas9, which is ∼1 kb smaller than Sp-Cas9, and our expanded-capacity and tropism-modified Gateway Ad5 vectors. While the PAM sequence of SaCas9 (NNGRRT) is more restrictive than Sp-Cas9 (NGG), the Sa-Cas9 PAM sequence still occurs once every 32 bp of random DNA, allowing for ample targeting locations throughout the genome (80). In addition, the modularity of pMAGIC allows future improvements and discoveries in Cas9 orthologs and effectors to be readily incorporated, as we demonstrated with the inclusion of the recently described x-Cas9(3.7) mutant (24). Previous applications of LSD1-based epigenome editing have utilized transient transfections (TALE-LSD1 (21)) and stable cell lines (Nm-dCas9/LSD1 (19)) making them unsuitable for broader applications. The unique features engineered into pMAGIC enabled us to perform a first-in-kind experiment involving the efficient delivery of Sa-dCas9/LSD1 into a primary cell type, the pancreatic islet β-cell, to functionally validate a role for Area IV in regulation of PDX1 expression. It should be noted that LSD1 has been suggested to be the sole epigenetic engineering tool capable of specific attenuation of enhancers with no effect on promoter activity (19). In contrast, KRAB fusions affect both enhancer and promoter activities (19,20), which may prove problematic based on the looping and close-proximity of enhancers and promoters in 3-dimensional space. Creation of pMAGIC now allows efficient delivery of the specific and potent dCas9/LSD1 fusion gene to a wide variety of disease-relevant cell types, including primary cells.

Due to our own studies on PDX1 (5,7,8,27), targeting Area IV was of interest based on the specificity of its activity in mature β-cells (25), and its inclusion in a region implicated in regulating glycemia (33), although the causal SNP(s) and corresponding elements have not yet been identified. Upon β-cell specific delivery of Sa-dCas9/LSD1 targeted to Area IV, we observed epigenetic alterations suggestive of enhancer attenuation in INS1 832/13 cells that corresponded with a significant decrease in PDX1 expression and significant modulation of classical PDX1 target genes in both INS1 832/13 cells and primary rat islets. While this manuscript was being prepared, Spaeth, *et al* (81), reported a mouse model in which Area IV was genetically deleted. In agreement with our findings, Area IV deletion resulted in a significant decrease in PDX1 expression, correlating with decreased β-cell function and replication. However, contrary to our findings, these phenotypes were only unveiled upon deletion of Area IV in the context of PDX1 heterozygosity, and then only in male mice (81). Our experiments, involving deliberate epigenetic modification of Area IV rather than its deletion, implicates the genomic region containing Area IV as having a more essential and fundamental role in maintaining proper PDX1 expression levels, even in the context of two functional PDX1 alleles. One explanation for the seemingly disparate findings lies in the difference between deleting an element versus engineering its epigenetic modification. Epigenetic profiling in human islets has found that the ∼0.5 kb Area IV element is part of a >10 kb stretch enhancer (13), and unpublished data from our group has found this stretch enhancer element is conserved in primary rat islets and INS1 832/13 cells. Therefore, the difference in these models may be a direct consequence of whether the function or higher order chromatin structure of the stretch enhancer encompassing Area IV has been perturbed. In fact, the degree to which individual regulatory elements contribute to overall stretch enhancer function is still unknown. It will be of particular interest in the future to apply the pMAGIC toolbox to the PDX1 locus in human islets, where SNPs encompassing this stretch enhancer have been associated with glycemic control. Our results suggest that modification of the highly conserved Area IV is sufficient to have significant impact on regulation of key β-cell genes. Overall, these results demonstrate the versatility, speed, and power of pMVP and pMAGIC to unravel the role of individual genes and genomic elements in control of gene expression and cellular biology.

## DATA AVAILABILITY

pMVP and pMAGIC components will be made available through Addgene.

## Supplementary Material

Supplementary Data
