# Supplementary material for: Creation of versatile cloning platforms for transgene expression and dCas9-based epigenome editing
Source: Nucleic Acids Res. 2018 Dec 27;47(4):e23. doi: 10.1093/nar/gky1286 (PMC6393299; doi:10.1093/nar/gky1286)
Supplement: Supplementary Data [file gky1286_supplemental_files.zip › Haldeman,etal.SupplementalFigure1.pptx]

## Slide 1
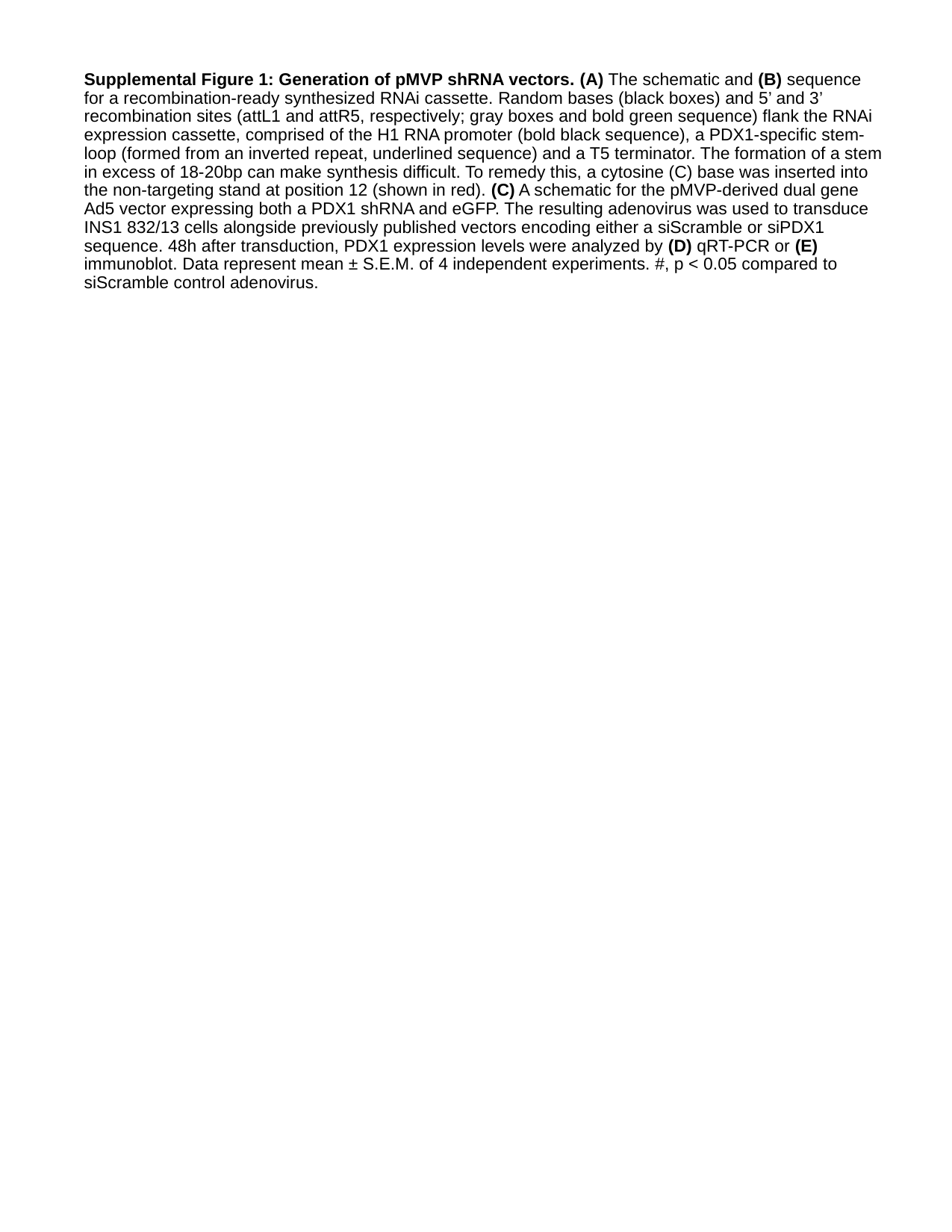

Supplemental Figure 1: Generation of pMVP shRNA vectors. (A) The schematic and (B) sequence for a recombination-ready synthesized RNAi cassette. Random bases (black boxes) and 5’ and 3’ recombination sites (attL1 and attR5, respectively; gray boxes and bold green sequence) flank the RNAi expression cassette, comprised of the H1 RNA promoter (bold black sequence), a PDX1-specific stem-loop (formed from an inverted repeat, underlined sequence) and a T5 terminator. The formation of a stem in excess of 18-20bp can make synthesis difficult. To remedy this, a cytosine (C) base was inserted into the non-targeting stand at position 12 (shown in red). (C) A schematic for the pMVP-derived dual gene Ad5 vector expressing both a PDX1 shRNA and eGFP. The resulting adenovirus was used to transduce INS1 832/13 cells alongside previously published vectors encoding either a siScramble or siPDX1 sequence. 48h after transduction, PDX1 expression levels were analyzed by (D) qRT-PCR or (E) immunoblot. Data represent mean ± S.E.M. of 4 independent experiments. #, p < 0.05 compared to siScramble control adenovirus.

## Slide 2
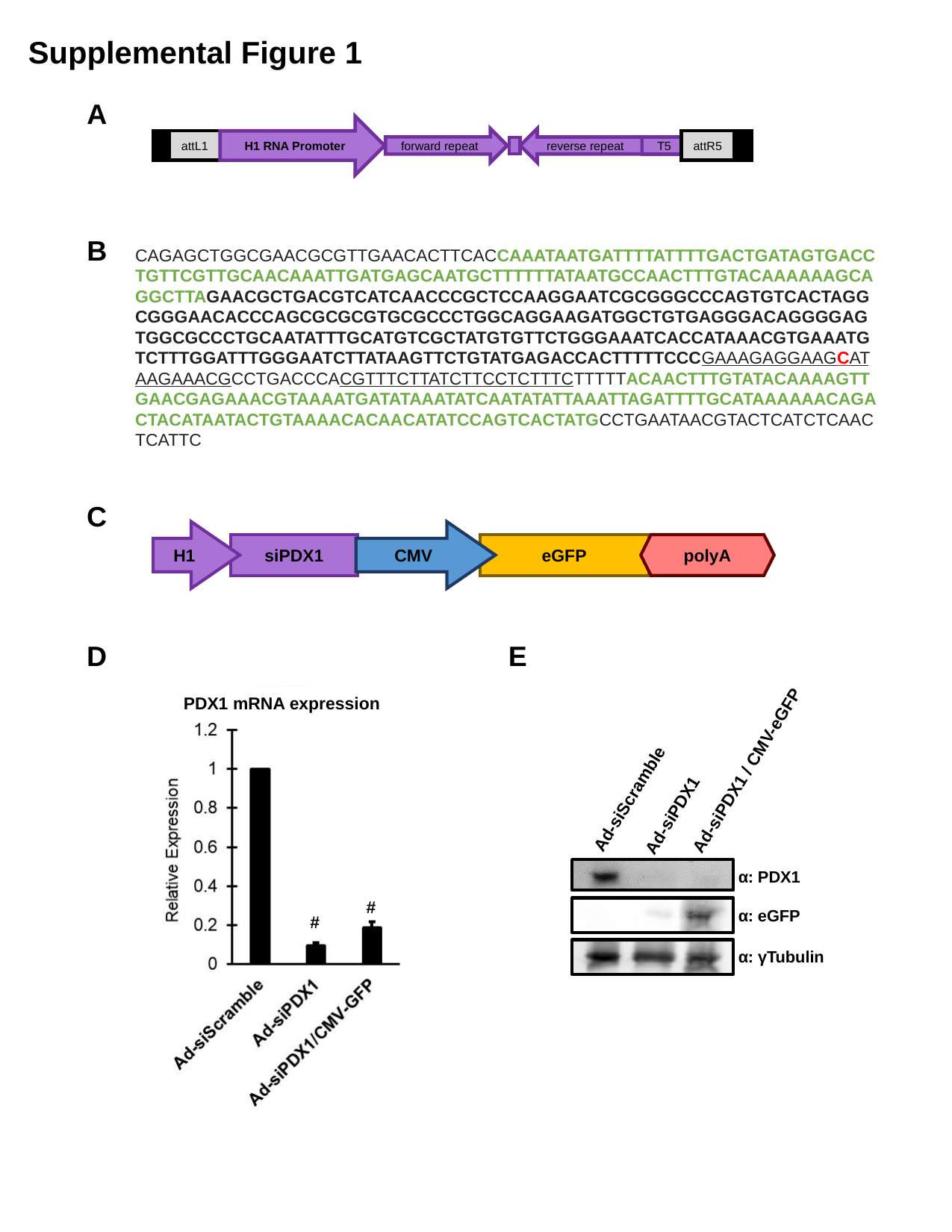

Supplemental Figure 1
A
H1 RNA Promoter
reverse repeat
attL1
attR5
T5
forward repeat
B
CAGAGCTGGCGAACGCGTTGAACACTTCACCAAATAATGATTTTATTTTGACTGATAGTGACCTGTTCGTTGCAACAAATTGATGAGCAATGCTTTTTTATAATGCCAACTTTGTACAAAAAAGCAGGCTTAGAACGCTGACGTCATCAACCCGCTCCAAGGAATCGCGGGCCCAGTGTCACTAGGCGGGAACACCCAGCGCGCGTGCGCCCTGGCAGGAAGATGGCTGTGAGGGACAGGGGAGTGGCGCCCTGCAATATTTGCATGTCGCTATGTGTTCTGGGAAATCACCATAAACGTGAAATGTCTTTGGATTTGGGAATCTTATAAGTTCTGTATGAGACCACTTTTTCCCGAAAGAGGAAGCATAAGAAACGCCTGACCCACGTTTCTTATCTTCCTCTTTCTTTTTACAACTTTGTATACAAAAGTTGAACGAGAAACGTAAAATGATATAAATATCAATATATTAAATTAGATTTTGCATAAAAAACAGACTACATAATACTGTAAAACACAACATATCCAGTCACTATGCCTGAATAACGTACTCATCTCAACTCATTC
C
H1
CMV
siPDX1
eGFP
polyA
D
E
PDX1 mRNA expression
Ad-siPDX1 / CMV-eGFP
Ad-siScramble
Ad-siPDX1
α: PDX1
#
α: eGFP
#
α: γTubulin
