# Supplementary material for: Creation of versatile cloning platforms for transgene expression and dCas9-based epigenome editing
Source: Nucleic Acids Res. 2018 Dec 27;47(4):e23. doi: 10.1093/nar/gky1286 (PMC6393299; doi:10.1093/nar/gky1286)
Supplement: Supplementary Data [file gky1286_supplemental_files.zip › Haldeman,etal.SupplementalFigure2.pptx]

## Slide 1
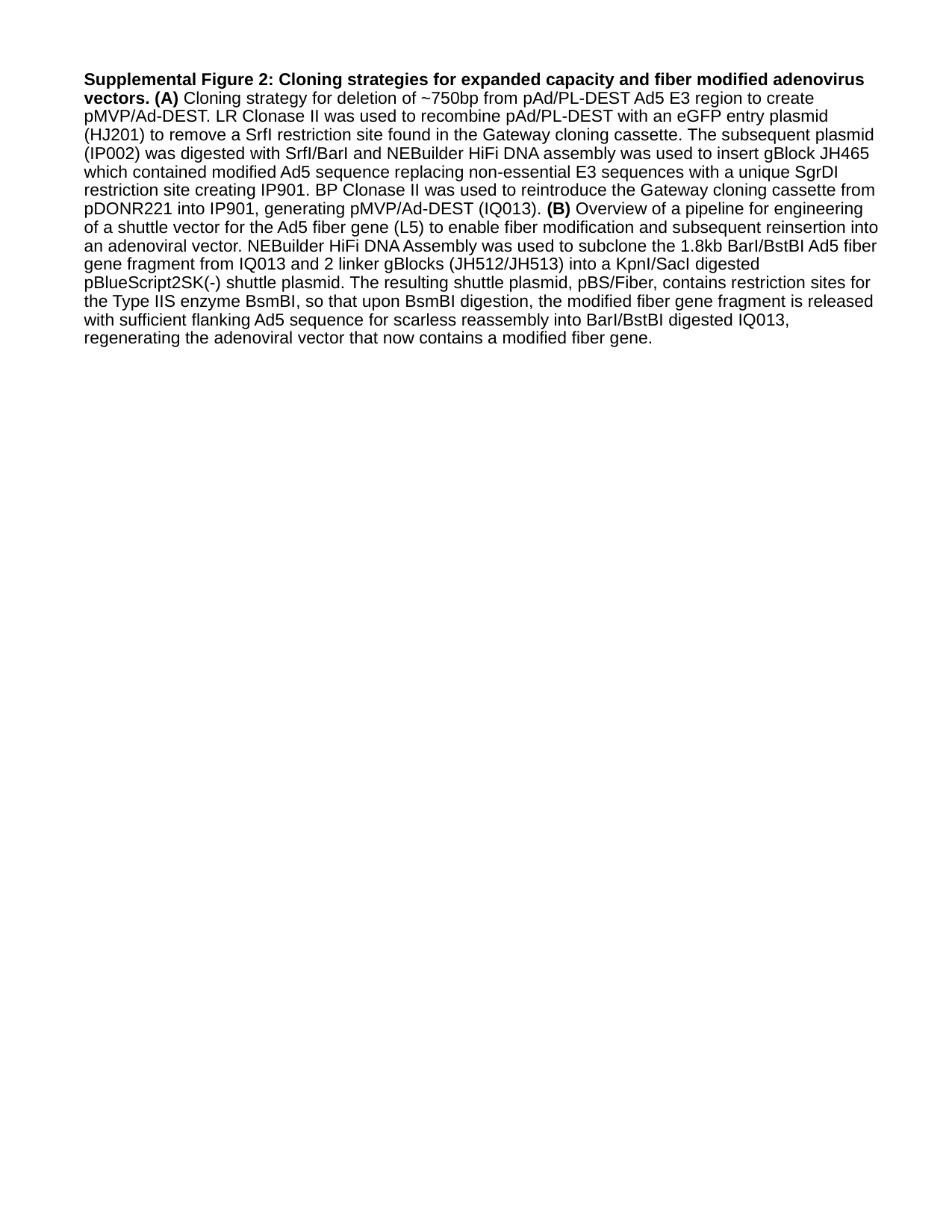

Supplemental Figure 2: Cloning strategies for expanded capacity and fiber modified adenovirus vectors. (A) Cloning strategy for deletion of ~750bp from pAd/PL-DEST Ad5 E3 region to create pMVP/Ad-DEST. LR Clonase II was used to recombine pAd/PL-DEST with an eGFP entry plasmid (HJ201) to remove a SrfI restriction site found in the Gateway cloning cassette. The subsequent plasmid (IP002) was digested with SrfI/BarI and NEBuilder HiFi DNA assembly was used to insert gBlock JH465 which contained modified Ad5 sequence replacing non-essential E3 sequences with a unique SgrDI restriction site creating IP901. BP Clonase II was used to reintroduce the Gateway cloning cassette from pDONR221 into IP901, generating pMVP/Ad-DEST (IQ013). (B) Overview of a pipeline for engineering of a shuttle vector for the Ad5 fiber gene (L5) to enable fiber modification and subsequent reinsertion into an adenoviral vector. NEBuilder HiFi DNA Assembly was used to subclone the 1.8kb BarI/BstBI Ad5 fiber gene fragment from IQ013 and 2 linker gBlocks (JH512/JH513) into a KpnI/SacI digested pBlueScript2SK(-) shuttle plasmid. The resulting shuttle plasmid, pBS/Fiber, contains restriction sites for the Type IIS enzyme BsmBI, so that upon BsmBI digestion, the modified fiber gene fragment is released with sufficient flanking Ad5 sequence for scarless reassembly into BarI/BstBI digested IQ013, regenerating the adenoviral vector that now contains a modified fiber gene.

## Slide 2
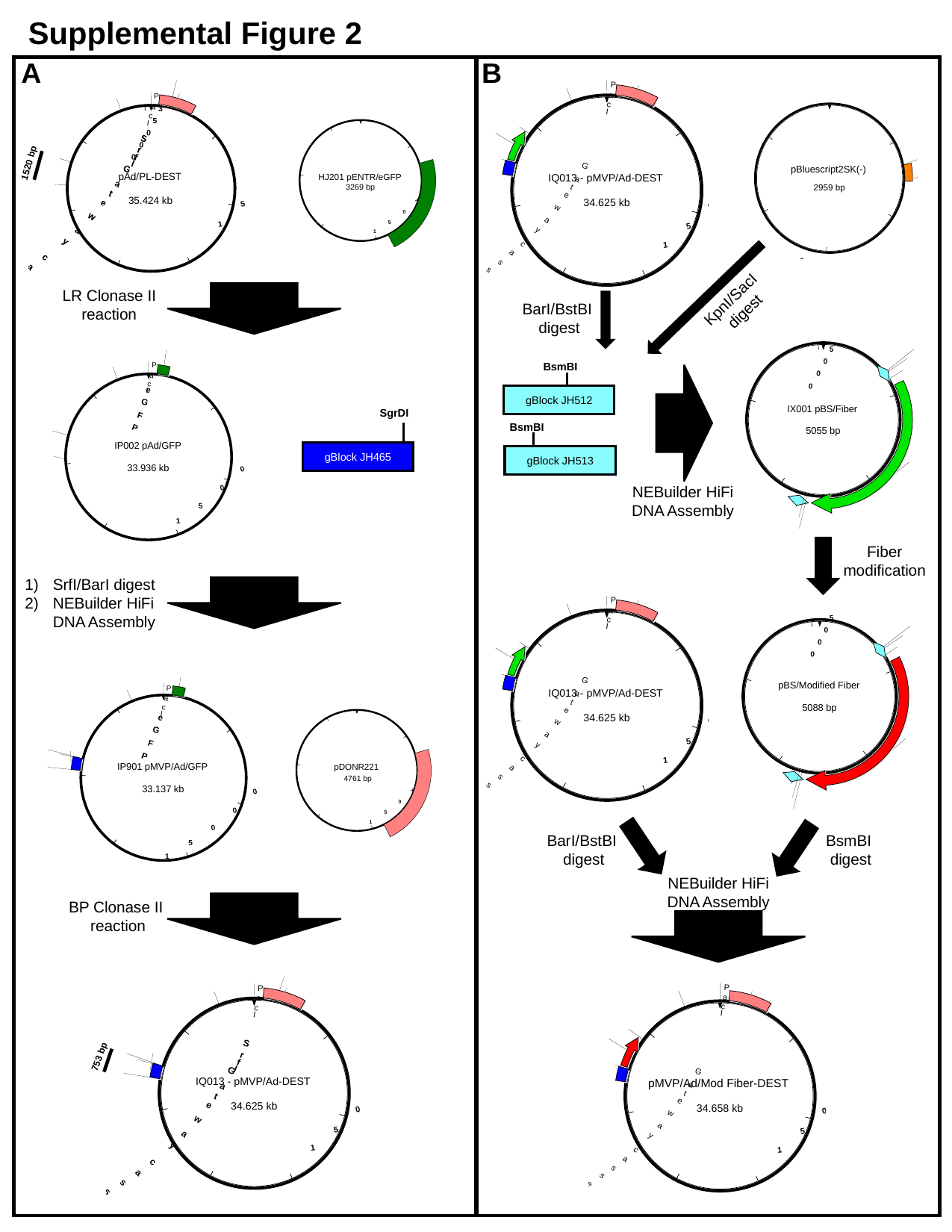

Supplemental Figure 2
A
B
1520 bp
KpnI/SacI
digest
LR Clonase II
reaction
BarI/BstBI
digest
BsmBI
gBlock JH512
BsmBI
gBlock JH513
NEBuilder HiFi DNA Assembly
SgrDI
gBlock JH465
Fiber modification
SrfI/BarI digest
NEBuilder HiFi DNA Assembly
4761 bp
BarI/BstBI
digest
BsmBI
digest
NEBuilder HiFi DNA Assembly
BP Clonase II
reaction
753 bp
