# Supplementary material for: Creation of versatile cloning platforms for transgene expression and dCas9-based epigenome editing
Source: Nucleic Acids Res. 2018 Dec 27;47(4):e23. doi: 10.1093/nar/gky1286 (PMC6393299; doi:10.1093/nar/gky1286)
Supplement: Supplementary Data [file gky1286_supplemental_files.zip › Haldeman,etal.SupplementalFigure3.pptx]

## Slide 1
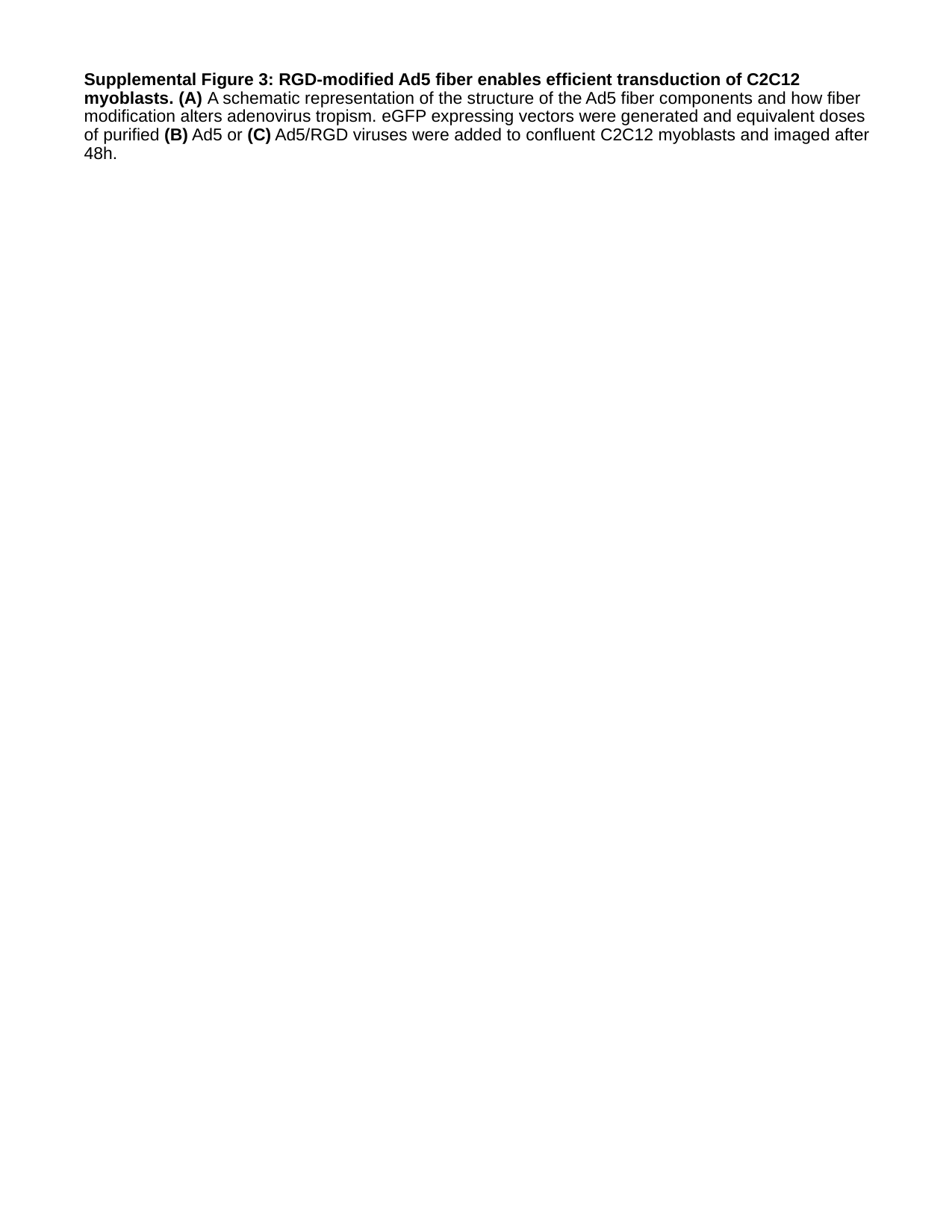

Supplemental Figure 3: RGD-modified Ad5 fiber enables efficient transduction of C2C12 myoblasts. (A) A schematic representation of the structure of the Ad5 fiber components and how fiber modification alters adenovirus tropism. eGFP expressing vectors were generated and equivalent doses of purified (B) Ad5 or (C) Ad5/RGD viruses were added to confluent C2C12 myoblasts and imaged after 48h.

## Slide 2
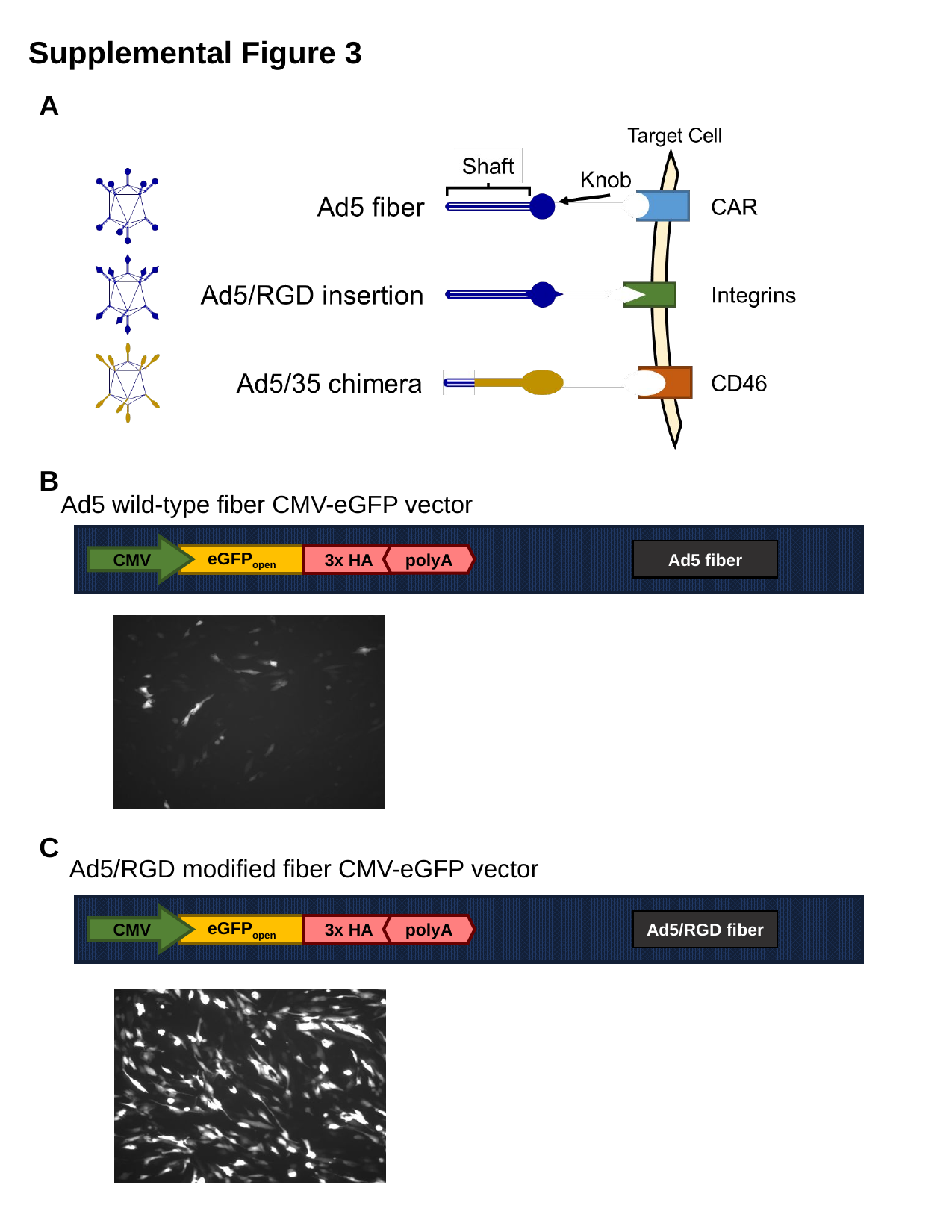

Supplemental Figure 3
A
B
Ad5 wild-type fiber CMV-eGFP vector
CMV
polyA
3x HA
eGFPopen
Ad5 fiber
C
Ad5/RGD modified fiber CMV-eGFP vector
CMV
polyA
3x HA
eGFPopen
Ad5/RGD fiber
