# Supplementary material for: Creation of versatile cloning platforms for transgene expression and dCas9-based epigenome editing
Source: Nucleic Acids Res. 2018 Dec 27;47(4):e23. doi: 10.1093/nar/gky1286 (PMC6393299; doi:10.1093/nar/gky1286)
Supplement: Supplementary Data [file gky1286_supplemental_files.zip › Haldeman,etal.SupplementalFigure4.pptx]

## Slide 1
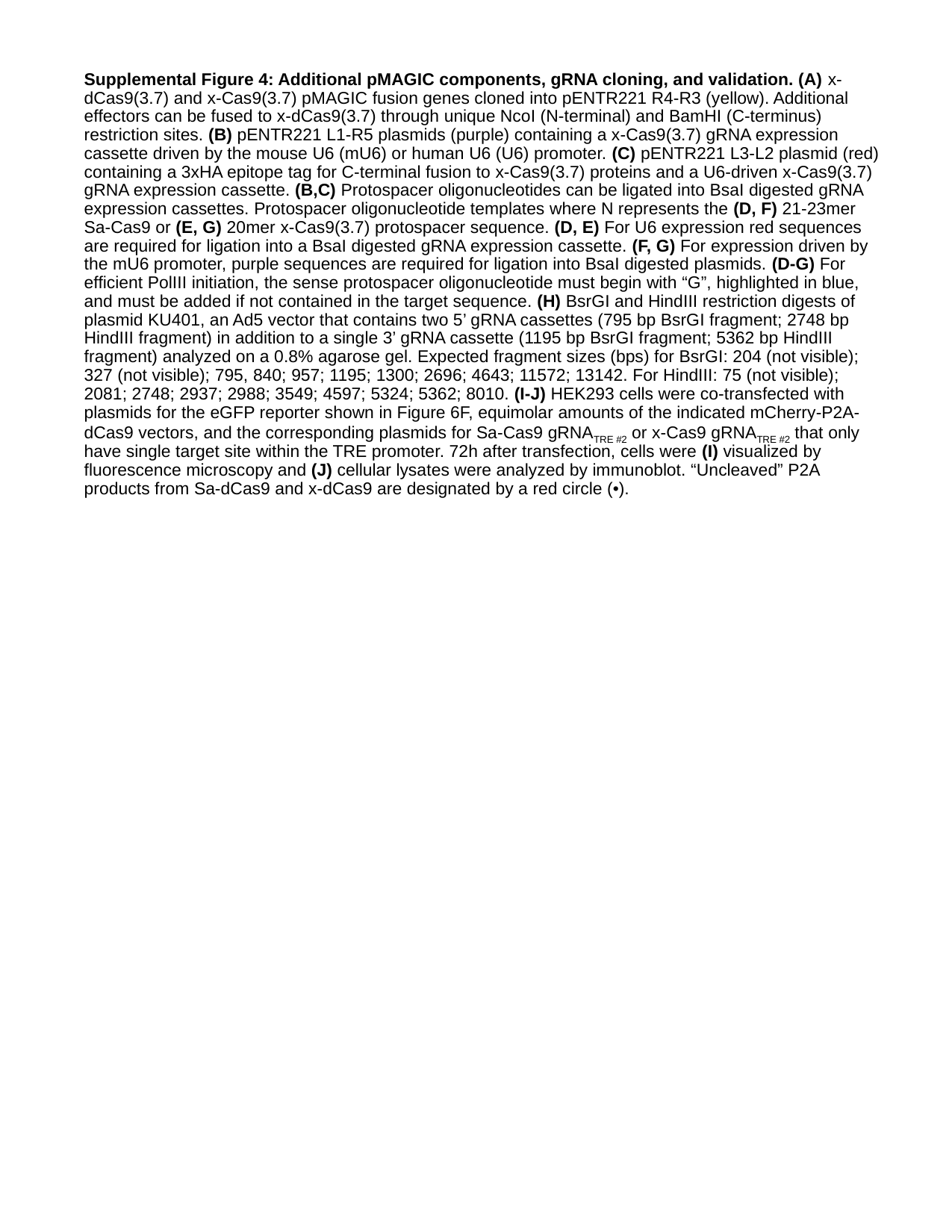

Supplemental Figure 4: Additional pMAGIC components, gRNA cloning, and validation. (A) x-dCas9(3.7) and x-Cas9(3.7) pMAGIC fusion genes cloned into pENTR221 R4-R3 (yellow). Additional effectors can be fused to x-dCas9(3.7) through unique NcoI (N-terminal) and BamHI (C-terminus) restriction sites. (B) pENTR221 L1-R5 plasmids (purple) containing a x-Cas9(3.7) gRNA expression cassette driven by the mouse U6 (mU6) or human U6 (U6) promoter. (C) pENTR221 L3-L2 plasmid (red) containing a 3xHA epitope tag for C-terminal fusion to x-Cas9(3.7) proteins and a U6-driven x-Cas9(3.7) gRNA expression cassette. (B,C) Protospacer oligonucleotides can be ligated into BsaI digested gRNA expression cassettes. Protospacer oligonucleotide templates where N represents the (D, F) 21-23mer Sa-Cas9 or (E, G) 20mer x-Cas9(3.7) protospacer sequence. (D, E) For U6 expression red sequences are required for ligation into a BsaI digested gRNA expression cassette. (F, G) For expression driven by the mU6 promoter, purple sequences are required for ligation into BsaI digested plasmids. (D-G) For efficient PolIII initiation, the sense protospacer oligonucleotide must begin with “G”, highlighted in blue, and must be added if not contained in the target sequence. (H) BsrGI and HindIII restriction digests of plasmid KU401, an Ad5 vector that contains two 5’ gRNA cassettes (795 bp BsrGI fragment; 2748 bp HindIII fragment) in addition to a single 3’ gRNA cassette (1195 bp BsrGI fragment; 5362 bp HindIII fragment) analyzed on a 0.8% agarose gel. Expected fragment sizes (bps) for BsrGI: 204 (not visible); 327 (not visible); 795, 840; 957; 1195; 1300; 2696; 4643; 11572; 13142. For HindIII: 75 (not visible); 2081; 2748; 2937; 2988; 3549; 4597; 5324; 5362; 8010. (I-J) HEK293 cells were co-transfected with plasmids for the eGFP reporter shown in Figure 6F, equimolar amounts of the indicated mCherry-P2A-dCas9 vectors, and the corresponding plasmids for Sa-Cas9 gRNATRE #2 or x-Cas9 gRNATRE #2 that only have single target site within the TRE promoter. 72h after transfection, cells were (I) visualized by fluorescence microscopy and (J) cellular lysates were analyzed by immunoblot. “Uncleaved” P2A products from Sa-dCas9 and x-dCas9 are designated by a red circle (•).

## Slide 2
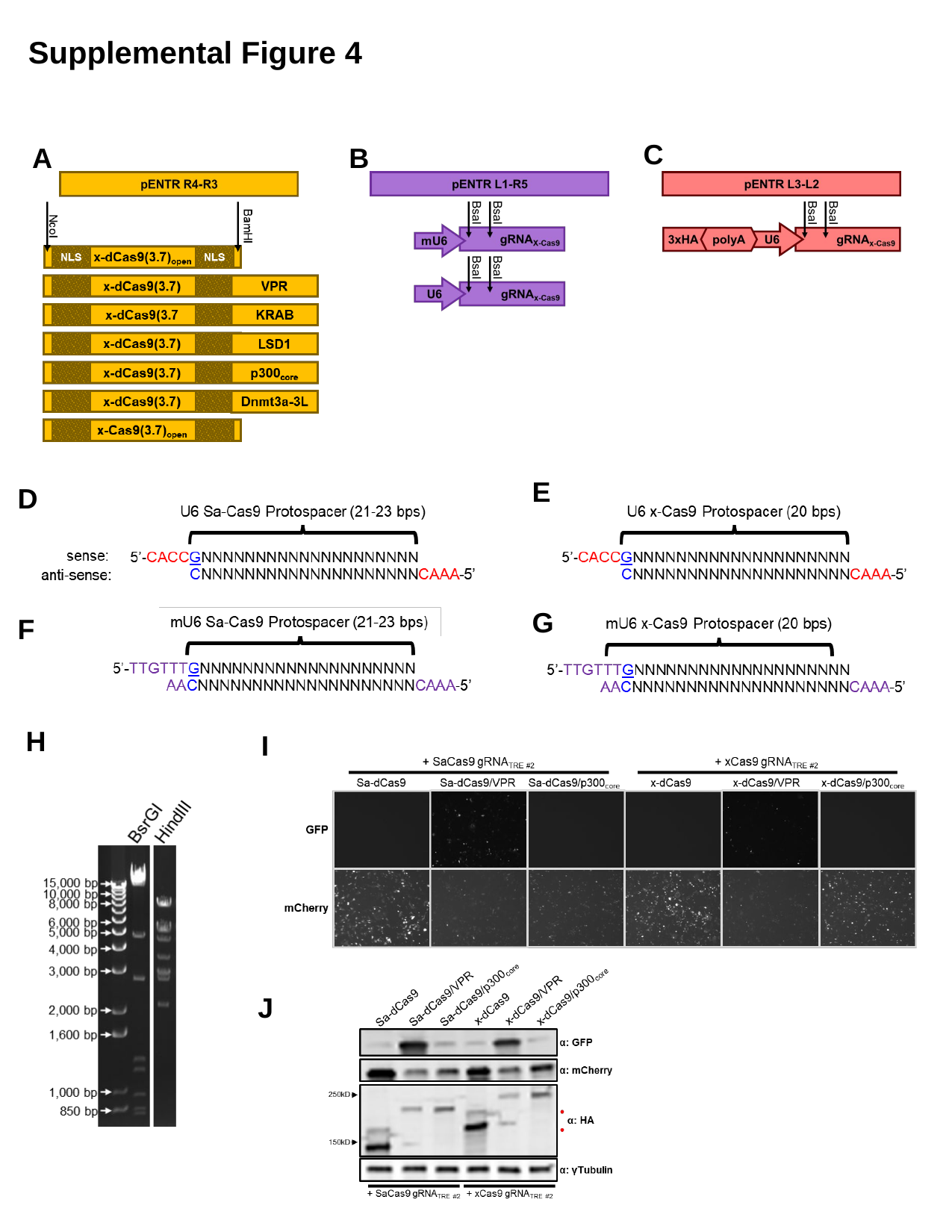

Supplemental Figure 4
C
A
B
E
D
G
F
H
I
J
