# Supplementary material for: Creation of versatile cloning platforms for transgene expression and dCas9-based epigenome editing
Source: Nucleic Acids Res. 2018 Dec 27;47(4):e23. doi: 10.1093/nar/gky1286 (PMC6393299; doi:10.1093/nar/gky1286)
Supplement: Supplementary Data [file gky1286_supplemental_files.zip › Haldeman,etal.SupplementalFigure5.pptx]

## Slide 1
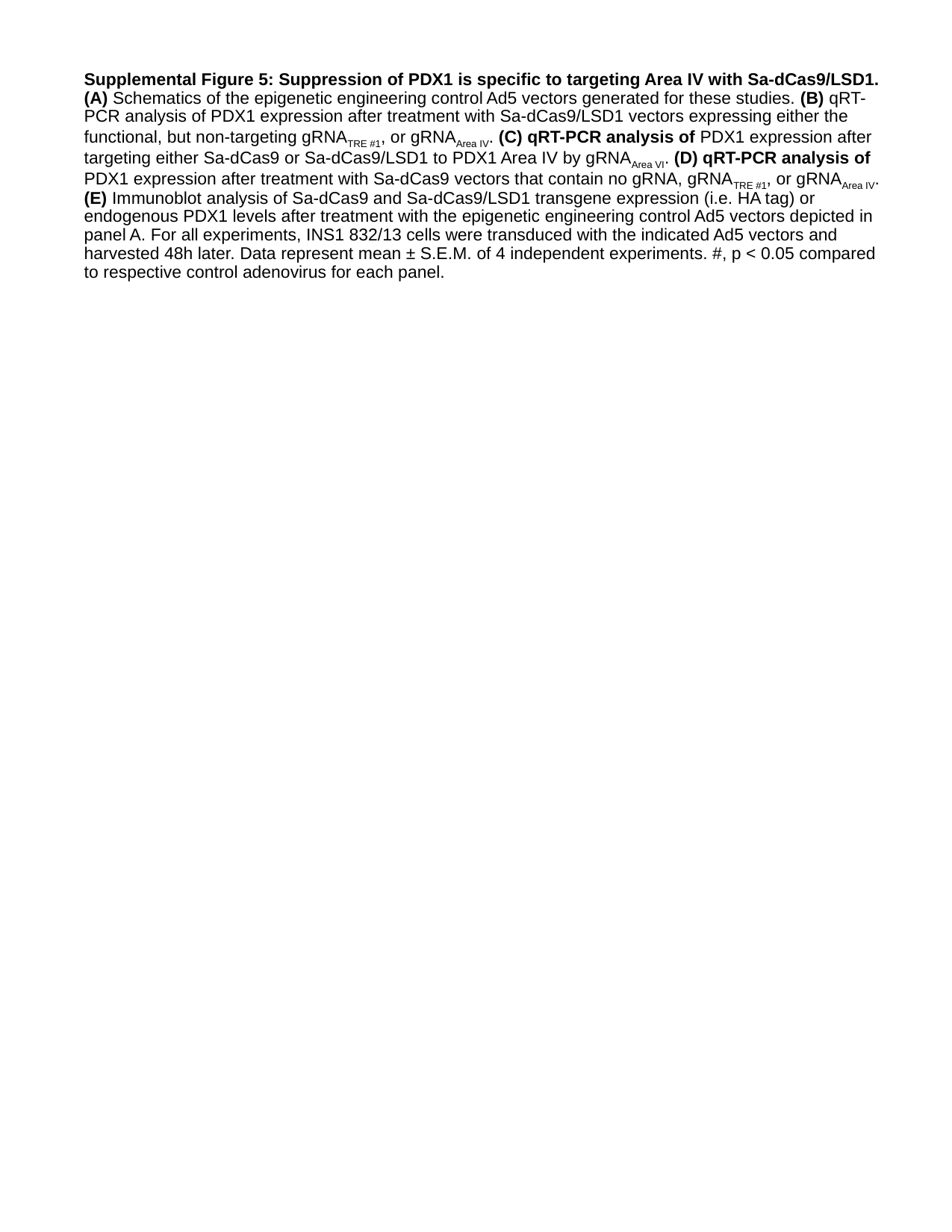

Supplemental Figure 5: Suppression of PDX1 is specific to targeting Area IV with Sa-dCas9/LSD1. (A) Schematics of the epigenetic engineering control Ad5 vectors generated for these studies. (B) qRT-PCR analysis of PDX1 expression after treatment with Sa-dCas9/LSD1 vectors expressing either the functional, but non-targeting gRNATRE #1, or gRNAArea IV. (C) qRT-PCR analysis of PDX1 expression after targeting either Sa-dCas9 or Sa-dCas9/LSD1 to PDX1 Area IV by gRNAArea VI. (D) qRT-PCR analysis of PDX1 expression after treatment with Sa-dCas9 vectors that contain no gRNA, gRNATRE #1, or gRNAArea IV. (E) Immunoblot analysis of Sa-dCas9 and Sa-dCas9/LSD1 transgene expression (i.e. HA tag) or endogenous PDX1 levels after treatment with the epigenetic engineering control Ad5 vectors depicted in panel A. For all experiments, INS1 832/13 cells were transduced with the indicated Ad5 vectors and harvested 48h later. Data represent mean ± S.E.M. of 4 independent experiments. #, p < 0.05 compared to respective control adenovirus for each panel.

## Slide 2
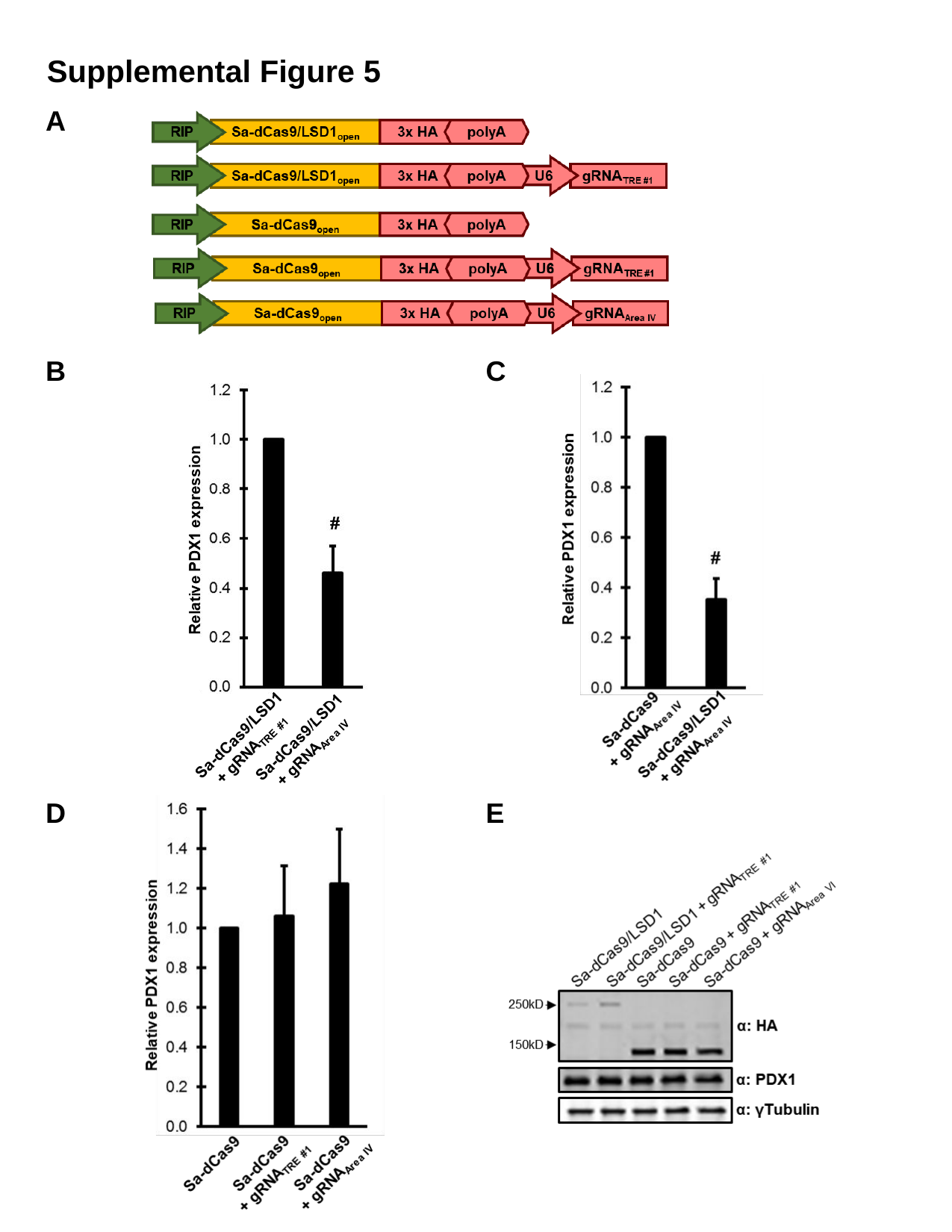

Supplemental Figure 5
A
B
C
D
E
